# Engineering an Alcohol-Forming Fatty Acyl-CoA Reductase for Aldehyde and Hydrocarbon Biosynthesis in *Saccharomyces cerevisiae*

**DOI:** 10.3389/fbioe.2020.585935

**Published:** 2020-10-06

**Authors:** Jee Loon Foo, Bahareh Haji Rasouliha, Adelia Vicanatalita Susanto, Susanna Su Jan Leong, Matthew Wook Chang

**Affiliations:** ^1^Department of Biochemistry, Yong Loo Lin School of Medicine, National University of Singapore, Singapore, Singapore; ^2^NUS Synthetic Biology for Clinical and Technological Innovation (SynCTI), National University of Singapore, Singapore, Singapore; ^3^Singapore Institute of Technology, Singapore, Singapore

**Keywords:** synthetic biology, metabolic engineering, protein engineering, *de novo* biosynthesis, biofuels, aldehydes

## Abstract

Aldehydes are a class of highly versatile chemicals that can undergo a wide range of chemical reactions and are in high demand as starting materials for chemical manufacturing. Biologically, fatty aldehydes can be produced from fatty acyl-CoA by the action of fatty acyl-CoA reductases. The aldehydes produced can be further converted enzymatically to other valuable derivatives. Thus, metabolic engineering of microorganisms for biosynthesizing aldehydes and their derivatives could provide an economical and sustainable platform for key aldehyde precursor production and subsequent conversion to various value-added chemicals. *Saccharomyces cerevisiae* is an excellent host for this purpose because it is a robust organism that has been used extensively for industrial biochemical production. However, fatty acyl-CoA-dependent aldehyde-forming enzymes expressed in *S. cerevisiae* thus far have extremely low activities, hence limiting direct utilization of fatty acyl-CoA as substrate for aldehyde biosynthesis. Toward overcoming this challenge, we successfully engineered an alcohol-forming fatty acyl-CoA reductase for aldehyde production through rational design. We further improved aldehyde production through strain engineering by deleting competing pathways and increasing substrate availability. Subsequently, we demonstrated alkane and alkene production as one of the many possible applications of the aldehyde-producing strain. Overall, by protein engineering of a fatty acyl-CoA reductase to alter its activity and metabolic engineering of *S. cerevisiae*, we generated strains with the highest reported cytosolic aliphatic aldehyde and alkane/alkene production to date in *S. cerevisiae* from fatty acyl-CoA.

## Introduction

Fatty aldehydes are a class of compounds with a wide range of applications, such as fragrances and flavorings (Kohlpaintner et al., 2013). Importantly, due to the reactivity of the carbonyl functional group, they are versatile chemicals that can undergo a wide range of reactions, including oxidation, reduction, addition, imination, and amination (Murray, 2019). Therefore, fatty aldehydes can be converted to a gamut of compounds and are important precursors in the chemical manufacturing industry (Kohlpaintner et al., 2013; Murray, 2019). Conventionally, fatty aldehydes and their derivatives are synthesized chemically from fossil resources, which require harsh conditions and expensive and/or toxic catalysts (Kohlpaintner et al., 2013). Alternatively, fatty aldehydes can be biosynthesized under ambient conditions from fatty acids or their acyl-CoA forms via enzymatic reactions in biological systems (Reiser and Somerville, 1997; Koeduka et al., 2002; Schirmer et al., 2010; Akhtar et al., 2013). The aldehydes could also serve as precursors to concurrently produce their derivatives *in vivo* via other metabolic pathways (Schirmer et al., 2010; Jin et al., 2016; Ladkau et al., 2016). Thus, metabolic engineering of microorganisms for biosynthesizing fatty aldehydes could provide a platform for sustainable and economical production of aldehydes from renewable resources. By introducing synthetic metabolic pathways, the aldehydes formed could also serve as substrates for conversion to a variety of valuable chemicals.

Initial successes in microbial fatty aldehyde bioproduction were achieved in *Escherichia coli* by employing fatty acyl-CoA reductase (FACR) or fatty acyl-(acyl-carrier-protein) (ACP) reductase (FAAR) to transform endogenous fatty acyl-CoAs and/or fatty acyl-A CPs to aldehydes (Reiser and Somerville, 1997; Schirmer et al., 2010). The aldehyde-producing microbes were applied in the context of biofuel production, as aliphatic and olefinic aldehydes can be transformed by aldehyde deformylating oxygenases (ADOs) or aldehyde decarbonylases (ADs) into alkanes and alkenes (ALKs) (Schirmer et al., 2010; Marsh and Waugh, 2013), which are ideal biofuel candidates since they are major components in fossil fuels and have high energy density. Subsequently, there was much interest in employing similar fatty acyl-CoA-dependent pathways for fatty aldehyde and ALK production in the model yeast *Saccharomyces cerevisiae* because it is a robust industrial host able to withstand harsh fermentation conditions and does not succumb to phage contamination (Hong and Nielsen, 2012; Foo et al., 2017). However, due to the poor activity of the aldehyde-forming FAARs and FACRs when used in the yeast strain (Buijs et al., 2014; Zhou et al., 2016b), fatty aldehyde production levels in *S. cerevisiae* were extremely low, leading to mediocre ALK production titers compared with those achieved in *E. coli* (Choi and Lee, 2013). Consequently, free fatty acid (FFA)-dependent pathways were preferred for fatty aldehyde and ALK production in *S. cerevisiae* because carboxylic acid reductase (CAR) and fatty acid α-dioxygenase (DOX) show higher activity in *S. cerevisiae* and produced more aldehydes as substrates for conversion to ALKs (Zhou et al., 2016b; Foo et al., 2017).

In this work, we sought to generate a catalytically efficient aldehyde-forming FACR to re-establish the feasibility of *de novo* fatty acyl-CoA-dependent ALK biosynthesis pathway in *S. cerevisiae* due to the merits of utilizing fatty acyl-CoAs as substrates. First, fatty acyl-CoA is readily available in *S. cerevisiae* for utilization in fatty acyl-CoA-dependent pathways without the need to overexpress thioesterases and delete fatty acyl-CoA synthetases to accumulate FFAs, which are required when implementing FFA-dependent pathways (Runguphan and Keasling, 2014). Second, the coenzyme A moiety is hydrophilic and possesses both acidic and basic functional groups. Therefore, fatty acyl-CoAs are much more soluble over a wider range of pH to serve as substrates than FFAs (Forneris and Mattevi, 2008), which are soluble only at high pH. Third, fatty acyl-CoAs are intracellular, while FFAs upon formation can diffuse or be transported out of the cells, often resulting in under-utilization of FFAs and resource wastage due to challenges in transporting extracellular FFAs back into the cells (Teixeira et al., 2017). Although no catalytically efficient aldehyde-forming FACR has been identified for application in *S. cerevisiae*, high levels of fatty alcohols have been produced in *S. cerevisiae* using heterologous alcohol-forming FACRs (Runguphan and Keasling, 2014; Feng et al., 2015; Zhou et al., 2016a). Hence, we aim to repurpose alcohol-forming FACR for aldehyde production by protein engineering.

**FIGURE 1:**
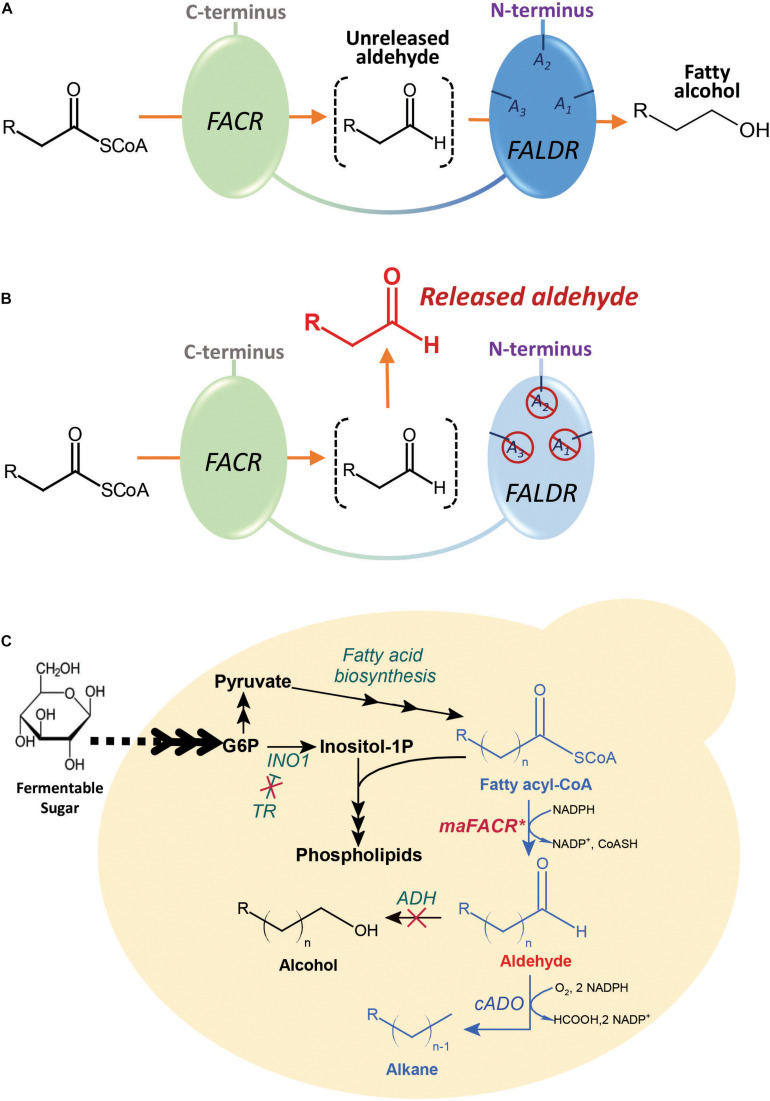
Schematic illustration of the maFACR engineering strategy and metabolic pathway for the fatty acyl-CoA-dependent production of alkanes and alkenes (ALKs) in engineered *Saccharomyces cerevisiae*. **(A)** maFACR converts fatty acyl-CoA to alcohol without releasing the aldehyde intermediate. It putatively has two distinct domains: a fatty acyl-CoA reductase (FACR) domain for reducing fatty acyl-CoA to aldehyde and a fatty aldehyde reductase (FALDR) domain to reduce aldehyde to fatty alcohol. The FALDR domain has catalytic residues A1, A2, and A3, which are found in this work to be Ser126, Tyr152, and Lys156, respectively. **(B)** Mutating the FALDR catalytic residues A1, A2, and A3 with the S126D, Y152F, and K156A modifications, respectively, inactivates the domain, thus allowing the release of aldehyde from the enzyme. **(C)** The engineered aldehyde-forming maFACR* is employed for conversion of endogenous fatty acyl-CoAs to aldehydes in *S. cerevisiae* and subsequent production of ALKs by deformylation of the aldehydes with cADO. To improve aldehyde titer, a transcription regulator (TR) was deleted to increase fatty acyl-CoA production, and ADHs were inactivated to diminish reduction of aldehydes to alcohols. *INO1*, inositol-3-phosphate synthase.

Alcohol-forming FACRs possess two reductase functions: one for reduction of fatty acyl-CoAs to aldehydes and another for subsequent reduction of aldehydes to alcohols. Although many of these FACRs have only one active site for both reductase functions (Hellenbrand et al., 2011), an alcohol-forming FACR from *Marinobacter aquaeolei* VT8, maFACR, was predicted to have two distinct domains, each putatively performing one reductase function (Willis et al., 2011; Figure 1A). Moreover, functional expression of maFACR in *S. cerevisiae* has been reported for fatty alcohol production (d’Espaux et al., 2017). Therefore, maFACR is a good candidate for rational engineering into an aldehyde-forming FACR by inactivating the domain that reduces aldehyde to alcohol (Figure 1B). Herein, we described identification of the catalytic residues of maFACR and verification of the two domains’ functions. Subsequently, maFACR was engineered into an aldehyde-forming FACR by inactivating the aldehyde reductase domain through mutation of the corresponding catalytic residues. *In vivo* production of aldehyde in *S. cerevisiae* was demonstrated using the engineered maFACR, and the production host was optimized to improve the aldehyde titer by increasing fatty acyl-CoA availability and deleting competing pathways. To exemplify application of the engineered maFACR for pathway construction in *S. cerevisiae*, the engineered maFACR was co-expressed with a cyanobacterial ADO (cADO) to achieve *de novo* production of ALK (Figure 1C). Upon optimization of the culture condition and expression system, we attained the highest reported cytosolic production of fatty aldehyde and ALK from fatty acyl-CoA in *S. cerevisiae* reported to date.

## Materials and Methods

### Strains, Oligonucleotides, Chemicals, and Culture Media

*Saccharomyces cerevisiae* BY4741 [American Type Culture Collection (ATCC)] was used to construct the yeast strains in this study. *Escherichia coli* TOP10 (Invitrogen) and Rosetta 2(DE3) (Novagen) were used for plasmid propagation, and protein expression and purification, respectively. Yeast extract, peptone, and tryptone were procured from BD Biosciences (Singapore). Molecular biology reagents were purchased from New England Biolabs (Singapore). Plasmids were isolated using QIAprep Spin Miniprep Kit (Qiagen). PCR purification and DNA gel extraction were performed with Wizard SV Gel and PCR Clean-Up System (Promega). The genes for maFACR [National Center for Biotechnology Information (NCBI) Protein ID: WP_011785966.1] from *Marinobacter aquaeolei* VT8 and cADO from *Synechococcus* elongatus PCC 7942 (NCBI Protein ID: WP_011378104.1) were obtained through gene synthesis (Genscript, China) and provided as plasmids pUC57-maFACR and pUC57-cADO, respectively. The sequences were codon-optimized for expression in *S. cerevisiae* and had the Kozak sequence AAAA added before the start codon of the genes. All other chemicals were purchased from Sigma Aldrich (Singapore) unless otherwise stated. All plasmids and yeast strains used in this study are listed in Supplementary Table S1. Oligonucleotides were synthesized by Integrated DNA Technologies (Singapore) and listed in Supplementary Table S2. All genes were verified by sequencing (1st BASE, Singapore) after cloning. Codon-optimized gene sequences are listed in Supplementary Table S6.

*E. coli* was cultivated in lysogeny broth (LB; 1% tryptone, 0.5% yeast extract, and 1% NaCl) and supplemented with ampicillin (100 mg/L) and/or chloramphenicol (30 mg/L) when required. YPD medium (1% yeast extract, 2% peptone, and 2% glucose) was used for non-selective cultivation of *S. cerevisiae*. Yeast transformants with URA3 and/or LEU2 selection markers were cultivated in yeast minimal medium consisting of yeast nitrogen base (YNB, 6.7 g/L) supplemented with the appropriate synthetic complete amino acid dropout mixture (YNB-URA, YNB-LEU, or YNB-URA-LEU), and glucose and/or galactose at required concentrations as carbon source. Solid growth media were similarly prepared with addition of 2% agar to the recipe described.

### Sequence Alignment of maFACR

Eight proteins were randomly selected from the MupV_like_SDR_e and SDR_c families using the NCBI database (Sayers et al., 2020), and their amino acid sequences were aligned to that of maFACR using ClustalX2 (Larkin et al., 2007).

### Protein Structure Homology Modeling and Analysis of maFACR

The amino acid sequence of maFACR was uploaded to the Robetta server (Kim et al., 2004). Pymol (Schrodinger, 2010) was used to align the five predicted structures and generate their rendered images.

### DNA Transformation and Strain Construction

Yeast competent cells were prepared, and DNAs were transformed using the LiOAc/PEG method (Gietz and Schiestl, 2007). *ADH1*–7 and *SFA1* were deleted from BY4741 using gene disruption cassettes as described in literature (Yu et al., 2016). Generation of BYΔ6 derivatives with multi-gene deletion was mediated by an adapted CRISPR/Cas9 system (Jakociunas et al., 2015) using protocols detailed in Supplementary Material.

### Plasmid Construction

#### Plasmid pmaFACR

maFACR was digested from pUC57-maFACR with *Hin*dIII/*Xho*I and cloned into pYES2/CT (Thermo Fisher, Singapore) to obtain pmaFACR.

#### Generation of maFACR Variants

Single-site mutants of maFACR were generated by QuikChange protocol (Agilent) using pmaFACR as template and complementary primer pairs, as indicated in Supplementary Table S2. Multi-site mutants were created by sequential mutation using the same protocol. maFACR with truncated N-terminal domain was generated by amplifying maFACR with the primer pair Nter-F/Nter-R. Similarly, the C-terminal domain was truncated from maFACR by amplifying maFACR with the primers Cter-F/Cter-R. The truncated maFACR genes were digested with *Hin*dIII/*Xho*I and ligated to pYES2/CT to create the single-domain forms of maFACR. The names of the variants and the respective mutations/truncations are denoted in Table 1.

**TABLE 1:** *In vitro* assay of FACR and FALDR activities in maFACR variants.

**maFACR variant^*a*^**	**FACR specific activity^*b*^**		**FALDR specific activity^*c*^**	
	**(nmol NTB min^–1^ mg^–1^ protein)**	**(nmol NTB min^–1^ nmol^–1^ protein)**	**(nmol NADP+ min^–1^ mg^–1^ protein)**	**(nmol NADP+ min^–1^ nmol^–1^ protein)**
maFACR_*WT*_	63.2 ± 1.5	7.3 ± 0.2	7789.3 ± 43.8	905.3 ± 5.1
maFACR_*S12*__6D_	60.9 ± 8.5	7.1 ± 1.0	ND	ND
maFACR_*K15*__6A_	73.2 ± 2.3	8.5 ± 0.3	ND	ND
maFACR_*Y15*__2F_	64.1 ± 0.9	7.4 ± 0.1	ND	ND
maFACR_*SYK*_	70.2 ± 0.8	8.2 ± 0.1	ND	ND
maFACR_*Cter*_	3.0 ± 0.2	0.23 ± 0.02	ND	ND
maFACR_*S51*__5A_	ND	ND	2,594.0 ± 285.0	301.5 ± 33.1
maFACR_*K52*__7A_	ND	ND	2,689.0 ± 73.1	312.5 ± 8.5
maFACR_*Y53*__2F_	ND	ND	2,440.6 ± 263.1	283.7 ± 30.6
maFACR_*Nter*_	ND	ND	12,875.0 ± 291.2	1,104.2 ± 25.0

*^*a*^maFACR_*WT*_ is the wild-type enzyme. maFACR_*SYK*_ has the mutations S126D, Y152F, and K156A. maFACR_*Nter*_ and maFACR_*Cter*_ are the N- and C-terminal domains, respectively. Otherwise, maFACR_*X*_ refers to a single-site mutant, whereby the mutation X is S126D, Y152F, K156A, S515A, Y527F, or K532A.^*b*^Palmitoyl-CoA was the substrate used.^*c*^Decanal was the substrate used.ND, not detected; FACR, acyl-CoA reductase; FALDR, fatty aldehyde reductase.*

#### Plasmid pMAL-maFACR and the Corresponding Plasmids for the Mutants

maFACR and the site-mutated variants were amplified with the primers maFACR-MAL-F/maFACR-MAL-R using the corresponding pYES2/CT-based plasmids as templates. The sequences encoding the N- and C-terminal domains of maFACR were subcloned by amplifying the respective genes from the corresponding pYES2/CT-based plasmids with the primer pairs maFACR-MAL-F/Nter-MAL-R and Cter-MAL-F/maFACR-MAL-R, respectively. The amplified genes were digested with *Ase*I/*Eco*RI and ligated with pMAL-c5x (New England Biolabs, Singapore) digested with *Nde*I/*Eco*RI.

#### Plasmids pUdTT and pUdAT

pUdTT and pUdAT were constructed by replacing the P_TEF__1_–P_GAL__1_ promoter cassette in pUdGT (Foo et al., 2017) with P_TEF__1_–P_TPI__1_ and P_TEF__1_–P_ADH__2_, respectively. P_TEF__1_ was amplified with the primer pairs pESC-pmt-TEF1-F/pESC-pmt-TEF1-R using pUdGT as template. P_TPI__1_ and P_ADH__2_ were amplified using the primer pairs pESC-pmt-TPI1-F/pESC-pmt-TPI1-R and pESC-pmt-ADH2-F/pESC-pmt-ADH2-R, respectively, and purified genomic DNA of BY4741 as template. The P_TEF__1_–P_TPI__1_ promoter cassette was generated by splicing P_TEF__1_ and P_TPI__1_ PCR fragments through overlap extension PCR using the primers pESC-pmt-TEF1-R and pESC-pmt-TPI1-R. The P_TEF__1_–P_ADH__2_ promoter cassette was created similarly using the P_ADH__2_ instead of P_TPI__1_ PCR fragment and pESC-pmt-ADH2-R instead of pESC-pmt-TPI1-R for overlap extension PCR. The P_TEF__1_–P_GAL__1_ and P_TEF__1_–P_ADH__2_ cassettes were digested with *Bam*HI/*Eco*RI and cloned into pUdGT to replace the P_TEF__1_–P_GAL__1_ segment, thus generating pUdTT and pUdAT, respectively.

#### Plasmids pUdGT-cADO, pUdTT-cADO, and pUdAT-cADO

cADO gene was digested from pUdGT-DOX-cADO (Foo et al., 2017) with *Bam*HI/*Xho*I and ligated into pUdGT, pUdTT, and pUdAT to obtain pUdGT-cADO, pUdTT-cADO, and pUdAT-cADO, respectively.

#### Plasmid pGT-ALK, pTT-ALK, and pAT-ALK

maFACR_*SYK*_ was amplified with the primer pair pESC-maFACR-F/pESC-maFACR-R from pmaFACR. The amplicon was digested with *Eco*RI/*Sac*I and ligated into pUdGT-cADO, pUdTT-cADO, and pUdAT-cADO to generate pGT-ALK, pTT-ALK, and pAT-ALK, respectively (Supplementary Figure S1).

### Protein Expression and Purification

maFACR and its mutants were expressed and purified by adaptation of the protocols in literature (Willis et al., 2011). *E. coli* Rosetta 2(DE3) harboring pMAL-maFACR was cultivated in 5 ml of LB with ampicillin and chloramphenicol (LBAC) overnight with shaking at 37°C. These starter cultures were diluted to OD_600_ ∼ 0.05 in 500 ml of fresh LBAC and grown to OD_600_ ∼ 0.5 with shaking at 37°C before the cells were induced with 200 μM of isopropyl-β-thiogalactopyranoside. The induced cells were grown at 16°C for 16 h with shaking at 225 rpm and harvested by centrifugation (4,000 × *g*, 5 min at 4°C). The cells were resuspended in 30 ml of chilled lysis buffer (20 mM of Tris–HCl, pH 7.0, 200 mM of NaCl, 1.0 mM of EDTA, and 10% glycerol) and passed thrice through a high pressure homogenizer (Avestin Emulsiflex C3, Germany) at 10,000 psi for lysis. The lysate was centrifuged (15,000 × *g*, 20 min at 4°C), and the soluble fraction was filtered through 0.45-μm filter. The filtrate was incubated with amylose beads (UcallM Biotechnology, China) for 30 min, and the mixture was loaded onto an Econo-Pac chromatography column (Bio-Rad, Singapore). The beads were washed 3 × 10 ml of binding buffer (20 mM of Tris–HCl, pH 7.0, 200 mM of NaCl, and 1.0 mM of EDTA) and 3 × 10 ml of equilibration buffer (20 mM of Tris–HCl, pH 7.0, and 50 mM of NaCl). The bound protein was eluted with 3 × 2 ml of elution buffer (20 mM of Tris–HCl, pH 7.0, 50 mM of NaCl, and 10 mM of maltose). All other maFACR mutants were similarly purified. The eluted proteins were buffer-exchanged with 3 × 15 ml of equilibration buffer in 100 kDa (for full-length maFACRs) or 50 kDa (for maFACR_*Cter*_ and maFACR_*Nter*_) cutoff ultrafiltration concentrator (Sartorius Vivaspin Turbo 15, Singapore) and concentrated to 0.5 ml. The extinction coefficients of the proteins were calculated by ProtParam in ExPASy (Gasteiger et al., 2003), and the concentration of the proteins was determined based on their absorption at 280 nm.

### *In vitro* Specific Activity Assays and Aldehyde Production Analysis

*In vitro* NADPH and 5,5’-dithiobis-(2-nitrobenzoic acid) (DTNB) specific activity assays were adapted from protocols in literature (Willis et al., 2011). A 4 × enzyme solution was prepared by diluting a maFACR variant in an assay buffer consisting of 80 mM of Tris–HCl (pH 7.0), 200 mM of NaCl, and 2 mg/ml bovine serum albumin (BSA). A 4 × palmitoyl-CoA substrate solution was prepared by diluting a 5 mM aqueous stock solution to 20 μM in deionized water. A 4 × decanal substrate solution was prepared by dissolving the aldehyde to 10 mM in dimethyl sulfoxide (DMSO) and diluting to 240 μM in deionized water. A 4 × DTNB solution was prepared by dissolving the reagent to 10 mg/ml in DMSO and diluting to 0.4 mg/ml in deionized water. A 4 × NADPH solution was prepared by dissolving the co-factor to 2 mg/ml in 1 mM of Tris–HCl, pH 7.0, and diluting to 0.6 mg/ml in deionized water. The FACR specific activity assays were performed by mixing 50 μl of the 4 × enzyme, palmitoyl-CoA, NADPH, and DTNB solutions in a 96-well plate and monitored at 340 nm on a Synergy HT multi-mode microplate reader (Biotek Instruments, Inc.). The fatty aldehyde reductase (FALDR) specific activity assays were similarly performed by replacing the palmitoyl-CoA and DTNB solutions with decanal and deionized water, respectively, and were monitored at 412 nm. Thus, the reaction mixtures contained 20 mM of Tris–HCl, pH 7.0, 200 mM of NaCl, 2 mg/ml of BSA, 0.5 mg/ml of NADPH, 60 μM of decanal or 5 μM of palmitoyl-CoA substrate, and 0.1 mg/ml of DTNB (for DTNB assay only). The final concentrations of the maFACR variants in the FACR and FALDR assays were 5.0 and 1.3 μM, respectively. All assays were performed in duplicates.

For *in vitro* aldehyde production analysis of maFAR_*SYK*_, 4 × substrate solutions of various chain lengths of fatty acyl-CoAs were prepared by diluting 5 mM of aqueous stock solutions to 100 μM in deionized water; 100 μl of reactions was prepared as described for the FACR assays, except that DTNB solution was replaced with deionized water. The reactions were incubated at 25°C for 18 h and extracted with 100 μl of ethyl acetate. The organic extracts were analyzed by gas chromatography–mass spectrometry (GCMS) as described in literature (Foo et al., 2017).

### *De novo* Biochemical Production and Analysis

All cultures were cultivated at 30°C with shaking at 225 rpm. Overnight starter cultures were prepared by growing the production strains in YNB-URA with 2.0% glucose (YD-U), and the cells were washed before being diluted to OD_600_ ∼ 0.4 in the respective media for biochemical production. For aldehyde production, the strains harboring pmaFACR or the various maFACR mutants were cultivated in 25 ml of YNB-URA with 0.2% glucose and 1.8% galactose. After 48 h of cultivation, the cells were harvested by centrifugation (3 min, 4,000 × *g*). For ALK production with pGT-ALK or pTT-ALK, strains transformed with the plasmids were cultivated in 50 ml of YDG-U (with varying concentrations of glucose and galactose) or YD-U, respectively. At specific time points, 10 ml of the cultures was harvested by centrifugation (3 min, 4,000 × *g*).

ALK production from BYΔ6OYGA with pAT-ALK by batch feeding with glucose was performed by starting the cell cultivation in 50 ml of YNB-URA with 0.8% glucose. After 24 h of growth and every subsequent 12 h between 24 and 72 h, 0.2% glucose was supplemented by addition of 0.5 ml of 20% glucose (total 2.0% glucose when corrected to 50 ml). Single feeding was performed similarly except that cell cultivation commenced in YD-U and 0.5 ml of sterile deionized water was added at each time point instead of 20% glucose. After 96 h of cultivation, 10 ml of the cultures was harvested by centrifugation (3 min, 4,000 × *g*). The harvested cells were processed and analyzed by GCMS as described in literature (Foo et al., 2017). All experiments were performed in biological duplicates.

## Results

### Identification of Domains and Catalytic Residues in maFACR

In order to engineer the alcohol-forming maFACR for aldehyde production, we sought to first analyze the protein sequence of maFACR to identify its domains and catalytic residues. A search using BLAST identified two distinct domains corresponding to short dehydrogenase/reductase (SDR) families, i.e., MupV_like_SDR_e family at the N-terminus and SDR_c family at the C-terminus. As reported by Willis et al. (2011), residues 370–660 at the C-terminus show high homology (74% similar and 53% identical) to residues 9–295 of an aldehyde-forming FACR from *Acinetobacter baylyi* (formerly *Acinetobacter calcoaceticus*). Therefore, the C-terminal domain possibly contributes to the FACR activity of maFACR for aldehyde biosynthesis from fatty acyl-CoA. By inference, since maFACR is an alcohol-forming FACR, it is hypothesized that the N-terminal domain functions as an aldehyde reductase to convert the aldehyde intermediate to alcohol, although this domain has only low homology to a known FALDR (Willis et al., 2011).

**FIGURE 2:**
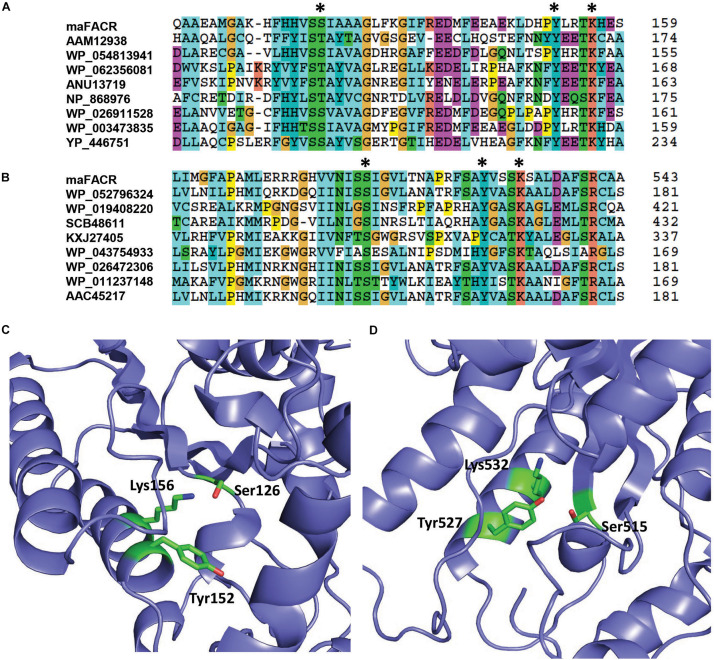
Identification of catalytic residues in maFACR. Alignments of the amino acid sequence of maFACR with proteins from the MupV_like_SDR_e and SDR_c families are shown in **(A,B)** to identify the catalytic residues (marked *) in the N- and C-terminal domains, respectively. The number on the right for each respective protein indicates the amino acid number of the last residue shown. The identified catalytic residues (in green) of the N- and C-terminal domains in a predicted structure of maFACR are shown in **(C,D)**, respectively.

Enzymes from SDR families are characterized by a serine/threonine–tyrosine–lysine catalytic triad (King et al., 2007). By aligning the maFACR sequence to that of proteins from the MupV_like_SDR_e and SDR_c families, Ser126, Tyr152, and Lys156 were identified as the catalytic residues of the N-terminal domain, while Ser515, Tyr527, and Lys532 were located as the catalytic residues of the C-terminal domain (Figures 2A,B). These are consistent with the orientation of the catalytic residues in the protein structures predicted by the Robetta server (Figures 2C,D and Supplementary Figure S2). In order to determine the enzymatic functions of the domains and verify the identities of the catalytic residues, we created variants of maFACR for *in vitro* assays. Specifically, the N- and C-terminal domains (i.e., maFACR_Nter_ and maFACR_Cter_, respectively) were expressed separately as residues 1–380 and 340–661, respectively, and the identified catalytic residues were inactivated with the following mutations: S126D, Y152F, and K156A in the N-terminal domain, and S515A, Y527F, and K532A in the C-terminal domain.

### Verification of Domain Functions and Catalytic Residues by *in vitro* Assay of maFACR Variants

*In vitro* assay of the FACR and FALDR activities of the maFACR variants was performed using palmitoyl-CoA and decanal, respectively, as substrates, because they were found to be the best substrates of maFACR (Willis et al., 2011). FACR activities of the maFACR variants were evaluated colorimetrically by using DTNB to measure the rate of CoASH liberated when fatty acyl-CoAs were converted to aldehydes. FALDR activities of the mutant enzymes were determined spectrometrically by monitoring the rate of NADPH depletion during reduction of aldehydes to alcohols. FACR and FALDR activities of wild-type maFACR were similar to those reported in literature (Willis et al., 2011; Table 1). Interestingly, the N-terminal domain maFACR_Nter_ showed 22.0% higher FALDR activity than wild-type maFACR (Table 1). In contrast, the C-terminal domain maFACR_Cter_ exhibited only 3.2% FACR activity as compared with the wild-type enzyme (Table 1). As predicted, no FALDR and FACR activities were detected from the C- and N-terminal domains, respectively. It is unclear why, relative to wild-type maFACR, maFACR_Nter_ has higher FALDR activity while maFACR_Cter_ has lower FACR activity, but it is known that protein truncation can alter structural flexibility, substrate accessibility, and quaternary structure of enzymes, which could all affect the activity (Gorfe et al., 2009; Yu et al., 2017; Latip et al., 2018). Nonetheless, these results strongly validate the hypothesis that the C-terminal domain is an FACR while the N-terminal domain functions as an FALDR.

To ascertain the catalytic residues, single-site S126D, Y152F, K156A, S515A, Y527F, and K532A mutants were assayed to establish their effects on the FACR and FALDR activities (Table 1). As expected, the mutations S126D, Y152F, and K156A in the N-terminal domain fully inactivated the FALDR activity of maFACR, while the FACR activity remained. Likewise, the FACR activity was abolished by the S515A, Y527F, and K532A mutations in the C-terminal domain, while the FALDR activity was still present, albeit lowered. These results verify that Ser126, Tyr152, and Lys156 are the catalytic residues for the FALDR activity in the N-terminal domain and that Ser515, Tyr527, and Lys532 are the catalytic residues for the FACR activity in the C-terminal domain. Furthermore, the triple-site S126D/Y152F/K156A mutant (maFACR_SYK_) converted C8–C18 fatty acyl-CoAs *in vitro* to aldehydes without detectable alcohols (Supplementary Figure S3), consistent with the broad fatty acyl-CoA substrate range of the wild-type maFACR reported (Willis et al., 2011). The absence of alcohol production by the triple-site mutant further indicates that the C-terminal domain has no FALDR activity and demonstrates the successful engineering of the alcohol-forming maFACR to one that produces aldehydes.

### *In vivo* Production of Fatty Aldehydes From Fatty Acyl-CoA With maFACR Variants and Engineered *Saccharomyces cerevisiae* Strains

**FIGURE 3:**
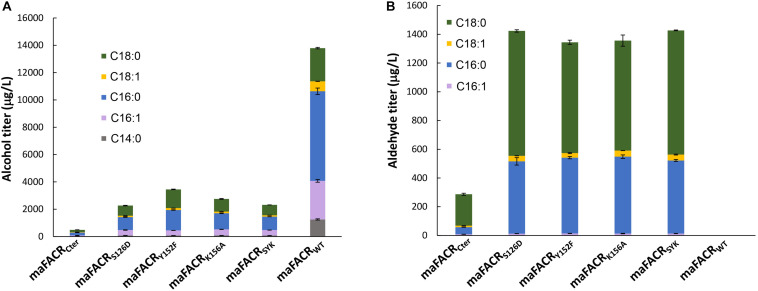
Fatty aldehyde and alcohol production by maFACR variants in *Saccharomyces cerevisiae*. Fatty aldehyde and alcohol production titers from *S. cerevisiae* expressing variants of maFACR are shown in **(A,B)**, respectively. Wild-type maFACR did not produce detectable fatty aldehyde. Data are shown as the mean ± SD of biological duplicates.

The wild-type maFACR and its aldehyde-forming variants were overexpressed in *Saccharomyces cerevisiae* for *in vivo* production of fatty aldehyde from endogenous fatty acyl-CoA. The maFACRs were all functionally expressed, as evidenced by the production of aldehydes and/or alcohols (Figure 3 and Supplementary Figure S4). As expected, the wild-type maFACR produced only alcohols and no detectable aldehydes (Willis et al., 2011). *S. cerevisiae* strains expressing the maFACR variants all produced aldehydes as well as alcohols, which were likely due to reduction of aldehydes by endogenous alcohol dehydrogenases (ADHs) (de Smidt et al., 2008), since we have demonstrated *in vitro* that the C-terminal domain of maFACR_SYK_ has no FALDR activity (Supplementary Figure S3). Nevertheless, compared with the wild-type maFACR, the amount of alcohols formed by the maFACR variants was markedly reduced due to the loss of FALDR activity from the enzyme (Figure 3A).

maFACRs with single-site S126D, Y152F, or K156A mutation produced similar amounts of total fatty aldehydes (1,432, 1,344, and 1,356 μg/L, respectively) (Figure 3B). The major aldehydes formed were hexadecanal (503–535 μg/L) and octadecanal (765–868 μg/L), along with small amounts of 9-octadecenal (31–42 μg/L) and 9–hexadecenal (13–14 μg/L). Shorter aldehydes were not detected, but the presence of 1-tetradecanol suggests that tetradecanal was produced but reduced by endogenous ADHs. Although it is straightforward to simply use the C-terminal FACR domain for aldehyde production, maFACR_Cter_ produced the lowest amount of aldehydes (287 μg/L) among the maFACR variants, which is consistent with the *in vitro* results (Table 1). Therefore, the maFACRs with mutated catalytic residues were preferred for *in vivo* aldehyde production. Henceforth, the triple-site S126D/Y152F/K156A mutant maFACR_SYK_, which performed similarly to the single-site mutants in terms of aldehyde and alcohol biosynthesis, was used in subsequent experiments for aldehyde production.

**FIGURE 4:**
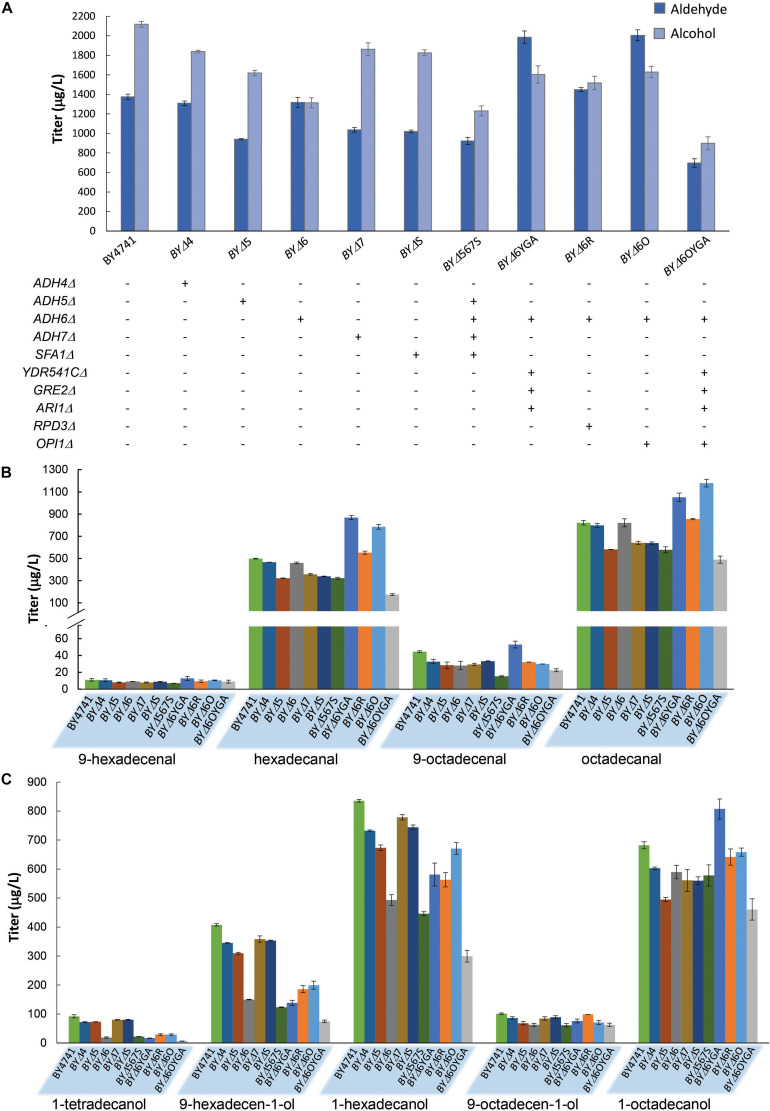
Fatty aldehyde and alcohol production from *Saccharomyces cerevisiae* mutants expressing maFACR_*SYK*_. **(A)** maFACR was expressed in *S. cerevisiae* mutants with various gene inactivation and the total fatty aldehyde and alcohol titers are shown. “+” and “-” denote the presence and absence of the indicated gene inactivation in the corresponding strain. **(B,C)** Show the compositions of fatty aldehydes and alcohols, respectively, in the various yeast mutants. Data are shown as the mean ± SD of biological duplicates.

In order to further improve aldehyde production, we attempted to first diminish aldehyde reduction to alcohols by deleting ADH genes. Eight widely studied ADHs, *ADH1*–7, and *SFA1* (de Smidt et al., 2008), were deleted to generate single-gene deletion strains for investigating the effects of the ADH deficiencies on aldehyde and alcohol production with maFACR_SYK_. Expression of maFACR_SYK_ in strains without *ADH1*, *ADH2*, or *ADH3* resulted in complete growth inhibition; thus, aldehyde and alcohol production could not be quantified. All other ADH deletion strains produced less alcohols than the wild-type strain (Figure 4A). Notably, total alcohol production was reduced most by *ADH6*Δ, from 2,118 μg/L in BY4741 to 1,314 μg/L in strain BYΔ6 (37.9% reduction). Being a medium-chain ADH (Larroy et al., 2002), *ADH6*Δ has greater effects on diminishing formation of shorter fatty alcohols, lowering levels of 1-tetradecanol, 9-hexadecen-1-ol, and 1-hexadecanol by 79.2, 63.2, and 41.0% while reducing production of 9-octadecenol and 1-octadecanol by 37.9 and 13.5%, respectively (Figure 4B). Despite the reduction in alcohol production, none of the deletions increased aldehyde production, although the *ADH4*Δ and *ADH6*Δ strains (i.e., BYΔ4 and BYΔ6, respectively) produced similar amounts of total aldehydes (1,310 and 1,318 μg/L, respectively) as compared with the parent strain (1,376 μg/L) (Figures 4A,C). Deletion of *ADH5*, *ADH7*, and *SFA1* lowered aldehyde production by at least 24%. In an attempt to further reduce formation of alcohols to accumulate aldehydes, particularly those shorter than C16, we further inactivated three ADHs that were reported to have a broad substrate range and activity on medium chain-length fatty aldehydes, i.e., *YDR541C*, *GRE2*, and *ARI1* (Liu and Moon, 2009; Moon and Liu, 2012, 2015), from the BYΔ6 strain. The 4-ADH-deficient strain, BYΔ6YGA, increased aldehyde production by 50.7% than did BYΔ6, reaching a titer of 1,986 μg/L (Figure 4A). Notably, although the amount of alcohols formed was increased to 1,603 μg/L, it was evidently lower than the level of aldehydes produced, essentially directing metabolic flux more toward the desired aldehydes than the alcohol side products.

Subsequently, we attempted to enhance aldehyde production by elevating substrate availability. Deletion of the transcription repressors *OPI1* and *RPD3* has been shown to increase fatty acyl-CoA biosynthesis for enhancing product titers in pathways that utilize fatty acyl-CoA as substrate (Teo et al., 2015). Hence, we disrupted *OPI1* and *RPD3* from the BYΔ6 strain (resulting in strains BYΔ6O and BYΔ6R, respectively) and overexpressed maFACR_SYK_ to determine the transcription repressor candidate that will benefit aldehyde production upon deletion. Both *OPI1*- and *RPD3*-disrupted strains increased aldehyde production, but deletion of *OPI1* conferred greater improvement, enhancing the aldehyde titer by 52.1% over the BYΔ6 strain (45.7% as compared with BY4741) to reach 2,005 μg/L (Figure 4A). Surprisingly, although BYΔ6O and BYΔ6YGA were the best aldehyde producers, combining the gene deletions to create strain BYΔ6OYGA was deleterious to aldehyde production, achieving a titer of only 697 μg/L. This is likely due to marked growth inhibition upon expressing maFACR_SYK_ in BYΔ6OYGA (Supplementary Figure S5). Nevertheless, we have demonstrated successful application of our engineered aldehyde-forming FACR for aldehyde production in *S. cerevisiae* and systematic host engineering for optimizing aldehyde titer.

### Application of maFAR_SYK_ for *de novo* Alkene Production From Fatty Acyl-CoA in *Saccharomyces cerevisiae*

**FIGURE 5:**
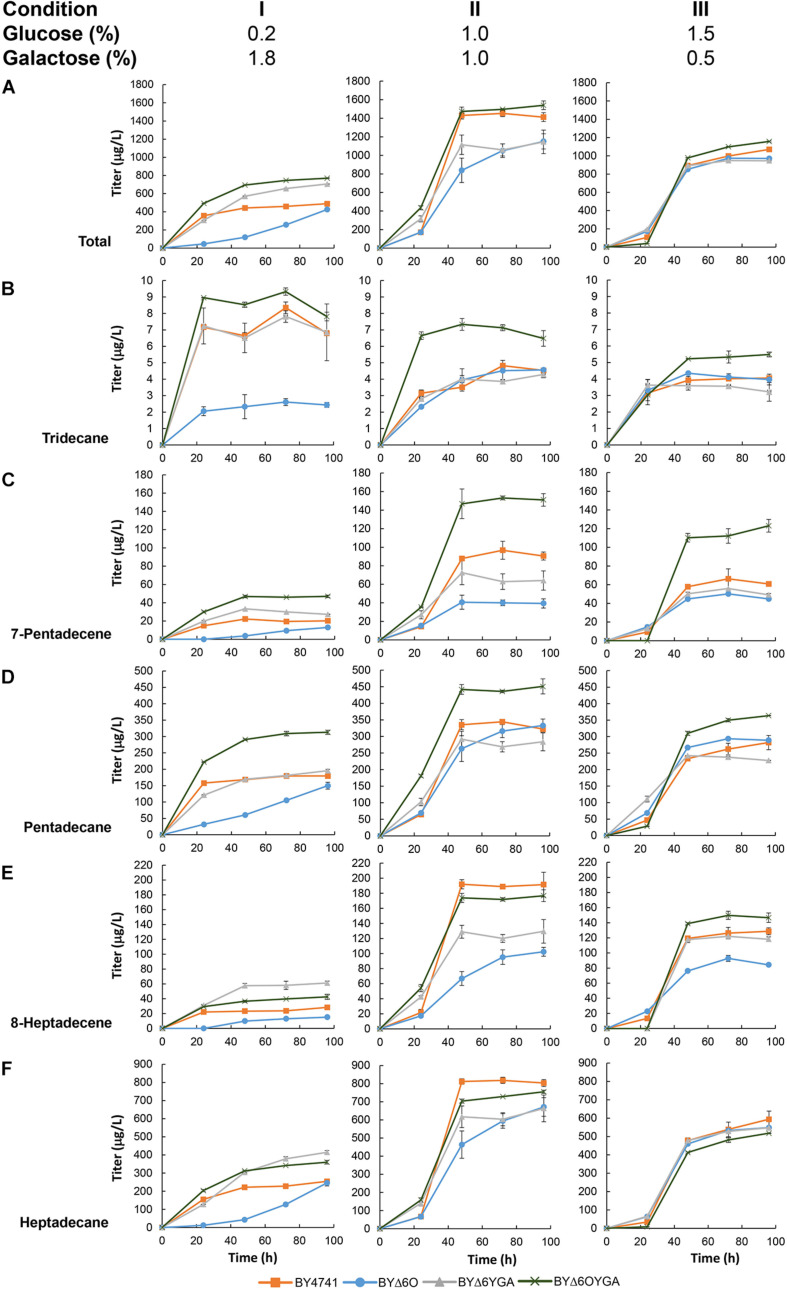
*De novo* alkane and alkene (ALK) production via a fatty acyl-CoA-dependent pathway in various *Saccharomyces cerevisiae* mutants. Time profiles of the ALK titers from different glucose/galactose ratio in BY4741, BYΔ6O, BYΔ6YGA, and BYΔ6OYGA harboring a fatty acyl-CoA-dependent ALK biosynthesis pathway are shown. Total titers **(A)** as well as the titers of individual ALK components, i.e., **(B)** tridecane, **(C)** 7-pentadecene, **(D)** pentadecane, **(E)** 8-heptadecene, and **(F)** heptadecane are presented. Data are shown as the mean ± SD of biological duplicates.

After establishing the capability of maFACR_SYK_ for aldehyde production in *S. cerevisiae*, we aimed to demonstrate the application of this enzyme for downstream production of biochemicals. By employing the aldehyde-forming maFACR_SYK_ and co-expressing a cADO from *Synechococcus elongatus* (Schirmer et al., 2010), we converted the saturated and unsaturated aldehydes formed to the biofuel candidates alkanes and alkenes, respectively, essentially achieving *de novo* production of ALKs from fermentable sugar (Figure 5 and Supplementary Figure S6A). We initially co-expressed both cADO with maFACR_SYK_ constitutively (under P_TEF__1_ and P_TPI__1_, respectively) with the pTT-ALK plasmid in BY4741 but were only able to produce 242 μg/L of ALKs (Supplementary Table S7). To improve the ALK titer, we utilized the plasmid pGT-ALK to express maFACR_SYK_ under the galactose-inducible P_GAL__1_ promoter instead, as we have demonstrated previously that controlled expression of the aldehyde-forming enzyme is beneficial for alkane production (Foo et al., 2017). By transforming pGT-ALK into BY4741, 489 μg/L of ALKs was produced in medium containing 0.2% glucose and 1.8% galactose after 96 h (Figure 5A, Condition I). Since the BYΔ6O and BYΔ6YGA host strains produced the highest amount of aldehydes, we attempted to improve ALK production in these strains. Indeed, ALK production was increased by 44.3% to 706 μg/L in BYΔ6YGA after 96 h. However, BYΔ6O/pGT-ALK exhibited a lag in growth and ALK production, reaching only 426 μg/L in titer after 96 h. Unexpectedly, although aldehyde production was not the highest in BYΔ6OYGA and BYΔ6OYGA/pGT-ALK exhibited growth inhibition, maximum ALK production reached 770 μg/L in BYΔ6OYGA, which is 9.2 and 57.5% higher than in BYΔ6YGA/pGT-ALK and BY4741/pGT-ALK, respectively. Interestingly, the improvement in maximum ALK titer in BYΔ6YGA/pGT-ALK over BY4741/pGT-ALK is mainly due to increased ALKs of longer chain lengths (63.1 and 116.0% increase in heptadecane and 8-heptadecene vs. 9.0 and 48.2% increase in pentadecane and 7-pentadecene, respectively, and 6.4% decrease in tridecane) (Figures 5B–F, Condition I). In contrast, the improved ALK production in BYΔ6OYGA/pGT-ALK is attributed to higher levels of shorter chain-length ALKs (11.6, 74.4, and 133.0% increase in tridecane, pentadecane, and 7-pentadecene vs. 41.5 and 50.5% increase in heptadecane and 8-heptadecene, respectively) (Figures 5B–F, Condition I).

To improve ALK production, we varied the proportion of glucose and galactose in the medium to improve cell growth and vary the induction of maFACR_SYK_ expression by galactose. Keeping total sugar concentration at 2.0%, glucose concentration was increased to formulate media with glucose/galactose ratio of 1.0%:1.0% and 1.5%:0.5%. Compared with cultivation in medium with 0.2% glucose, growth and ALK production of all strains improved when glucose concentration was increased to 1.0% (Figure 5A, Condition II and Supplementary Figure S6B). The greatest fold improvement in ALK production was found in BY4741/pGT-ALK, increasing the titer by 3.5-fold to achieve 1,496 μg/L. BYΔ6OYGA/pGT-ALK is still the highest producer, reaching a maximum ALK titer of 1,540 μg/L. Although the titers of BY4741/pGT-ALK and BYΔ6OYGA/pGT-ALK were almost identical, the chain-length profile of the strains was noticeably different, with BYΔ6OYGA/pGT-ALK again showing ability to produce more C13 and C15 ALKs than the other strains (Figures 5B–F, Condition II). Specifically, tridecane, pentadecane, and 7-pentadecene peak titers of BYΔ6OYGA/pGT-ALK were 52.0, 58.3, and 31.0% higher than BY4741/pGT-ALK, but heptadecane and 8-heptadecene peak titers of BYΔ6OYGA/pGT-ALK were 7.9 and 7.7% lower than BY4741/pGT-ALK, respectively. Further, increasing glucose concentration to 1.5% led to decrease in ALK production (Figure 5A, Condition III), possibly due to excessive repression of maFACR_SYK_ expression under the P_GAL__1_ promoter.

**FIGURE 6:**
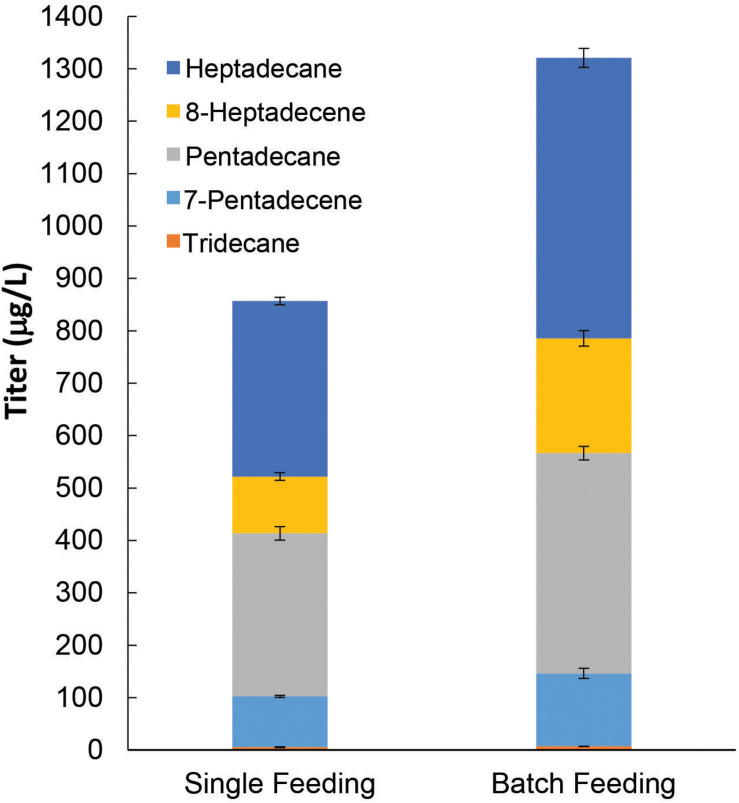
*De novo* alkane and alkene (ALK) production from fatty acyl-CoA via an inducer-independent biosynthetic pathway in *Saccharomyces cerevisiae*. By expressing maFACR_*SYK*_ under the growth phase-dependent P_*ADH*__2_ promoter, an inducer-independent ALK biosynthesis pathway with controlled expression of maFACR_*SYK*_ was implemented. In the single feeding experiment, 2% glucose was fed at the start of the cultivation. In the batch feeding experiment, 2% glucose was fed in batches over the course of the cultivation. Data are shown as the mean ± SD of biological duplicates.

Thus far, the ALK production pathway that we have constructed using pGT-ALK relies on galactose for induction of maFACR_SYK_ expression. However, galactose is much more expensive than glucose and is not economical, particularly for large-scale cultures. As we have shown that constitutive expression of maFACR_SYK_ from pTT-ALK is deleterious to ALK production, we therefore replaced the P_GAL__1_ promoter with the growth-phase-dependent P_ADH__2_ promoter to construct another plasmid, pAT-ALK. Using P_ADH__2_, maFACR_SYK_ expression is strongly repressed in the presence of glucose and will be expressed upon glucose depletion (Lee and DaSilva, 2005), thus effecting controlled expression of maFACR_SYK_ without the need for additional inducer. Hence, with pAT-ALK, we can implement the ALK biosynthesis pathway in *S. cerevisiae* using a medium with only glucose as the carbon source, which is a system that is more economical and industrially relevant than if galactose is required. The plasmid was transformed into our best host strain for ALK production, BYΔ6OYGA, and the resulting strain was cultivated in medium with 2.0% glucose supplied by single feeding or batch feeding for producing ALKs. In the single-feeding experiments, 2.0% glucose was provided at the start of the cultivation, and this produced 856.7 μg/L ALKs after 96 h (Figure 6). By supplying the 2.0% glucose through batch feeding, i.e., starting with 0.8% glucose and supplementing with 0.2% glucose every 12 h between 24 and 72 h, the final ALK titer was increased by 54.2% to 1,321 μg/L. The improved ALK titer using batch feeding of glucose might be due to reduced repression of maFACR_SYK_ expression since glucose concentration was kept lower throughout the cultivation compared with single feeding. This could have increased the availability of aldehyde for conversion to ALKs. Interestingly, the OD_600_ of the cultures after 96 h was higher when glucose was provided by batch feeding (OD_600_ = 14.5) than single feeding (OD_600_ = 10.1), suggesting that the Crabtree effect could have been reduced by batch feeding, which resulted in increased biomass and ALK production (Halka et al., 2018). Although the ALK titer achieved with BYΔ6OYGA/pAT-ALK by continuous glucose feeding was slightly lower (85.8%) than the highest ALK titer produced in BYΔ6OYGA/pGT-ALK under galactose-dependent condition (1,540 μg/L, Figure 5A, Condition II), it may be improved by optimizing the glucose concentration and feeding strategy during the fed-batch cultivation. Overall, we have demonstrated downstream application of our engineered maFACR_SYK_ for production of ALKs and enhanced the ALK biosynthesis by optimizing the culture composition and choice of promoters.

## Discussion

Aldehyde-forming bacterial enzymes have been employed with success for producing aldehydes in *Escherichia coli*, particularly the FACR from *Acinetobacter baylyi* and FAAR from *Synechococcus elongatus* (Schirmer et al., 2010; Lehtinen et al., 2018). However, there has been limited success in converting fatty acyl-CoAs into aldehydes in *Saccharomyces cerevisiae* due to the low activity of the aldehyde-forming FAARs and FACRs when employed in the yeast strain (Supplementary Figure S7; Buijs et al., 2014; Zhou et al., 2016b). In contrast, alcohol-forming bacterial, mammalian, and avian FACRs have been functionally expressed in *S. cerevisiae* for high-level fatty alcohol production (Runguphan and Keasling, 2014; Feng et al., 2015; d’Espaux et al., 2017). Analysis of the mammalian and avian FACRs shows only one distinct active site for reduction of both fatty acyl-CoA and aldehyde (Hellenbrand et al., 2011); thus, these enzymes are difficult to engineer rationally to eliminate solely the FALDR activity. On the other hand, maFACR has two distinct domains that appear to correspond to FACR and FALDR domains. Therefore, we selected this enzyme for rational engineering because the FALDR activity can be inactivated independent of the FACR activity. Indeed, we successfully repurposed in this work the alcohol-forming maFACR into one that is aldehyde-forming, thus demonstrating the importance of protein engineering for synthetic biology and metabolic engineering applications (Foo et al., 2012).

**FIGURE 7:**
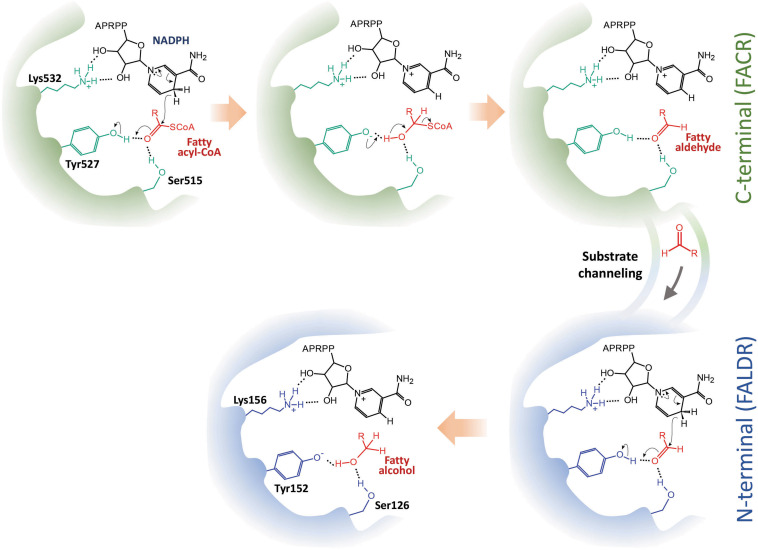
Proposed reaction mechanism of maFACR. We propose a mechanism whereby fatty acyl-CoA is reduced in the C-terminal fatty acyl-CoA reductase (FACR) domain (in green) to aldehyde, which is transferred to the N-terminal fatty aldehyde reductase (FALDR) domain (in blue) for further reduction to fatty alcohol. Ser515 and Tyr527 in the FACR domain first activate the carboxyl group of the fatty acyl-CoA by hydrogen bonding. NADPH, which interacts with Lys532 via hydrogen bonding, donates a hydride to reduce the fatty acyl-CoA to a hemithioacetal upon proton donation by Try527. Subsequent elimination of CoASH forms a fatty aldehyde, which is channeled to the N-terminal. Similarly, Ser126 and Tyr152 activate the carbonyl group of the fatty aldehyde to facilitate reduction by a hydride donated by an NADPH bound to Lys156. Upon accepting a proton from Tyr152, a fatty alcohol is formed. APRPP, adenosine-2-phosphate ribose pyrophosphate moiety of NAPDH.

Through *in vivo* enzymatic assay, we have verified the catalytic roles of the two domains of maFACR and identified the catalytic residues involved. The reduction of fatty acyl-CoA to fatty alcohol by maFACR was proposed to proceed via a reaction mechanism where a two-step reduction occurred within one active site or two highly cooperative active sites through a hemithioacetal intermediate covalently bound to maFACR (Willis et al., 2011). Our results indicate that two active sites are involved, whereby fatty acyl-CoA is reduced to aldehyde in the C-terminal domain and further reduced to alcohol in the N-terminal domain. Additionally, structures proposed by homology modeling of maFACR with the Robetta server do not show any cysteine near the catalytic residues (Supplementary Figure S8). Thus, an enzyme-bound thiohemiacetal intermediate appears to be unlikely. Since the two domains of maFACR belong to SDR families, we propose that each domain employs the general SDR catalytic mechanism involving the Ser-Tyr-Lys triad for substrate binding, hydride transfer, and co-factor binding (Figure 7; Nobutada et al., 2001). It is unclear how the aldehyde intermediate is transferred from the FACR domain to the FALDR domain without releasing the aldehyde from the enzyme, but efficient substrate channeling between domains/protomers has been well-documented in enzymes (Huang et al., 2001). The crystal structure of maFACR will be required to determine the exact mechanism for aldehyde transfer between the domains.

We employed the engineered maFACR_SYK_ for *in vivo* aldehyde production in *S. cerevisiae* BY4741 and was already able to produce 1,376 μg/L aldehyde without strain optimization. This contrasts with previous reports that deletion of the aldehyde dehydrogenase *HFD1* was critical for aldehyde production in *S. cerevisiae* by preventing oxidation of aldehydes to fatty acids (Buijs et al., 2014; Zhou et al., 2016a). Interestingly, deleting this gene from *ADH6*Δ was deleterious to aldehyde titer, drastically reducing the total aldehyde formed by 97.5–33 μg/L (Supplementary Table S8). This could be due to differences in strain background, as *HFD1*Δ in BY4741 also led to reduced titer when DOX from rice was used for aldehyde production (Foo et al., 2017). Thus, *HFD1* deletion was not investigated further. Nevertheless, we successfully improved BY4741 to increase aldehyde and reduce alcohol production by deleting several alcohol dehydrogenases and upregulating fatty acyl-CoA biosynthesis.

Although our efforts in this work have enhanced aldehyde biosynthesis in *S. cerevisiae*, there is still much room for improvement. To further increase aldehyde production, directed evolution of maFACR_SYK_ and other alcohol-producing FACRs may be explored to generate mutants with higher aldehyde-producing ability. Notably, TaFACR and MmFACR from owl and mouse, respectively, were shown to produce much more fatty alcohols than maFACR (d’Espaux et al., 2017), suggesting that these avian and mammalian FACRs have higher activities in converting fatty acyl-CoAs to aldehyde intermediates. However, as aforementioned, avian and mammalian FACRs may share the same active site for reduction of both fatty acyl-CoAs and aldehydes (Hellenbrand et al., 2011) and thus could not be easily engineered rationally like maFACR to eliminate the FALDR activity. Nevertheless, if directed evolution of TaFACR and MmFACR can significantly increase the affinity for fatty acyl-CoA over aldehyde, highly active aldehyde-forming variants can potentially be generated. Furthermore, despite deletion of several transcription regulators and ADHs, the improvement in aldehyde accumulation is still limited. One possible reason is the presence of several aldehyde dehydrogenases (ALDHs) in *S. cerevisiae* other than *HFD1*. Deletion of the five other major ALDHs (*ALD2–6*) (Navarro-Avino et al., 1999) may be evaluated to determine if enzymatic oxidation could be reduced to aid aldehyde accumulation. Expression of efflux pumps and the use of solvent overlay may also be investigated to transfer the aldehydes out of the cells to drive the flux toward aldehyde production by minimizing *in vivo* enzymatic conversion of aldehyde to by-products (Zhang et al., 2016; Zhou et al., 2016a; Perez-Garcia and Wendisch, 2018). The use of efflux pumps and solvent overlay has been successfully employed to improve biochemical production and hence may also be applicable for improving the accumulation of aldehydes (Zhang et al., 2016; Zhou et al., 2016a).

For ALK production, BYΔ6OYGA is the best host strain, although BYΔ6O and BYΔ6YGA are better host strains for producing aldehydes, suggesting a synergy between the deletion of *OPI1* along with the four ADHs that benefits the deformylation of aldehydes to ALKs, particularly those of shorter chain lengths. The reason is unclear, although it is possible that *OPI1* deletion increased the availability of fatty acyl-CoA, and deletion of the four medium-chain ADHs reduced competition from the ADHs with the cADO for the shorter chain-length aldehyde substrates, thus increasing the titer and skewing the ALK production profile toward shorter chain length. It is also noted that *YDR541C*, *GRE2*, and *ARI1* are NADPH-dependent ADHs (Liu and Moon, 2009; Choi et al., 2010; Moon and Liu, 2015). Therefore, their absence may improve availability of NADPH to cADO, which requires two molecules of NADPH for each deformylation reaction, hence accelerating the deformylation step. Further experiments will be required to elucidate the roles of the deletions in BYΔ6OYGA that promote ALK production. Nonetheless, we have achieved ALK titer up to 1,540 μg/L, which is to our knowledge the highest cytosolic ALK production to date in *S. cerevisiae* from fatty acyl-CoA. Even without genetic modification of the parent strain BY4741, our ALK production pathway using our engineered maFACR attained 1,496 μg/L ALK, which is already approximately 40- and 10-fold higher than the ALK titers reported in wild-type (Zhou et al., 2016a) and engineered *S. cerevisiae* strains (Zhou et al., 2016b), respectively, using cytosolic fatty acyl-CoA-based pathways with a low-activity aldehyde-forming FAAR (Table 2). In recent works on ALK production in *S. cerevisiae*, FFA-based pathways using CAR or DOX were favored for forming aldehydes toward ALK production due to the low activity of aldehyde-forming FACRs in *S. cerevisiae* (Zhou et al., 2016b; Foo et al., 2017). With our engineered maFACR_SYK_, we have achieved ALK titers that are comparable with those attained via FFA-dependent pathways, including those based on fatty acid decarboxylases (Chen et al., 2015; Zhu et al., 2017; Table 2), thus re-establishing the viability of the fatty acyl-CoA-based ALK production pathway. By employing maFACR_SYK_ in conjunction with novel strategies, such as organelle targeting of the ALK production pathway (Xu et al., 2016; Zhou et al., 2016a) and genetic circuit development (Lo et al., 2016; Xia et al., 2019), and expressing maFACR_SYK_ in non-conventional oleaginous host strains, such as *Yarrowia lipolytica* (Xu et al., 2016), ALK production in yeast can potentially be further improved. However, the ALK titers obtained in yeast strains still pale in comparison with those achieved in *E. coli* (Choi and Lee, 2013). More studies are required to identify the bottlenecks of ALK production pathways in yeast, such as competing pathways, co-factor availability, and low activity of cADO. As advances in synthetic biology and synthetic genomics for *S. cerevisiae* gain momentum (Chen et al., 2018; Foo and Chang, 2018), new tools are increasingly available for improving characteristics of yeast to maximize the potential of yeast as a production host for fatty aldehydes and their derivatives.

**TABLE 2:** Comparison of reported ALK production titers in yeast.

**Host**	**ALK pathway enzymes**	**Remarks**	**ALK titer (mg/L)^a^**	**References**
*Saccharomyces cerevisiae*	Engineered FACR and cADO	Wild-type *S. cerevisiae*	**1.496**	This work
	Engineered FACR and cADO	Four ADHs were deleted. Fatty acyl-CoA biosynthesis was upregulated by deleting *OPI1.*	**1.54**	
*S. cerevisiae*	Cyanobacterial FAAR and cADO	*HFD1* aldehyde dehydrogenase gene was deleted.	**0.14**	Zhou et al., 2016b
	CAR and cADO	*POX1* was deleted to inactivate beta-oxidation. *HFD1* and *ADH5* were deleted to inactivate competing pathways.	0.8	
*S. cerevisiae*	Cyanobacterial FAAR and cADO	Cytosolic ALK pathway in wild-type *S. cerevisiae.*	**0.035**	Zhou et al., 2016a
	CAR and cADO	Cytosolic ALK pathway in wild-type *S. cerevisiae.*	0.06	
	CAR and cADO	Cytosolic ALK pathway. *POX1* was deleted to inactivate beta-oxidation. *HFD1*, *ADH5*, and *SFA1* were deleted to inactivate competing pathways.	0.7	
	CAR and cADO	Peroxisomal ALK pathway. *POX1* was deleted to inactivate beta-oxidation. *HFD1* was deleted to inactivate competing pathway.	1.2	
	CAR and cADO	Peroxisomal ALK pathway. *POX1* was deleted to inactivate beta-oxidation. *HFD1* was deleted to inactivate competing pathway. Peroxisome biogenesis was increased by deleting *PEX31–32* and overexpressing *PEX34*.	3.55	
*S. cerevisiae*	DOX and cADO	*FAA1* and *FAA4* were deleted to accumulate FFAs.	0.074	Foo et al., 2017
*Yarrowia lipolytica*	Bacterial FACR and cADO	Cytosolic ALK pathway in an oleaginous yeast host.	**3.2**	Xu et al., 2016
	Bacterial FACR and cADO	The ALK pathway was targeted to the endoplasmic reticulum of the oleaginous yeast.	**16.8**	
	CAR and cADO	Cytosolic ALK pathway in an oleaginous yeast host.	23.3	
*S. cerevisiae*	Fatty acid decarboxylase, UndA	The host was engineered to produce medium-chain fatty acids and inactivate the beta-oxidation pathway. The highest titer was achieved with 20 g/L of glucose.	3.35	Zhu et al., 2017
*S. cerevisiae*	Fatty acid decarboxylase, OleT	*FAA1* and *FAA4* were deleted to accumulate FFAs. *HEM3* was overexpressed to increase the heme co-factor. *CCP1* was deleted to accumulate H_2_O_2_. The highest titer was achieved upon gene expression tuning and bioreactor process optimization.	3.7	Chen et al., 2015

*ALK, alkane and alkene; FACR, fatty acyl-CoA reductase; FAAR, fatty acyl-(acyl-carrier-protein) reductase; cADO, cyanobacterial aldehyde deformylating oxygenase; FFA, free fatty acid.^*a*^Titers of fatty acyl-CoA-dependent ALK pathway are shown in bold. Otherwise, the titers were from FFA-dependent ALK pathways.*

## Conclusion

In this work, we successfully engineered an alcohol-forming FACR into one that produces aldehyde and validated the functions of the two domains in the enzyme as well as the catalytic residues. By expressing the engineered maFACR_SYK_ in *Saccharomyces cerevisiae* and strain optimization through gene deletion to increase substrate availability and inactivate competing pathways, 2,005 μg/L of fatty aldehyde was produced. To our knowledge, this is the highest reported fatty aldehyde titer produced from fatty acyl-CoA in *S. cerevisiae*. Subsequently, we demonstrated the utilization of our engineered maFACR_SYK_ for downstream application, namely, ALK production, in this work. In combination with culture optimization, we attained ALK titer of 1,540 μg/L and skewed the ALK production profile toward shorter chain length. The ALK titer is the highest achieved to date via cytosolic ALK production in *S. cerevisiae* from fatty acyl-CoA. We believe that our engineered maFACR_SYK_ and yeast strains re-established the feasibility of aldehyde production from fatty acyl-CoA in *S. cerevisiae* for potential applications in biosynthesizing ALKs and other valuable aldehyde-derived compounds.

## Data Availability Statement

All datasets presented in this study are included in the article/Supplementary Material.

## Author Contributions

JF, BR, and AS performed the experiments and analyzed the experimental data. MC, SL, and JF oversaw the project and provided guidance. JF, BR, and MC wrote, reviewed, and edited the manuscript. All authors have read and agreed to the published version of the manuscript.

## Conflict of Interest

The authors declare that the research was conducted in the absence of any commercial or financial relationships that could be construed as a potential conflict of interest.
