# Supplementary material for: Engineering an Alcohol-Forming Fatty Acyl-CoA Reductase for Aldehyde and Hydrocarbon Biosynthesis in Saccharomyces cerevisiae
Source: Front Bioeng Biotechnol. 2020 Oct 6;8:585935. doi: 10.3389/fbioe.2020.585935 (PMC7573125; doi:10.3389/fbioe.2020.585935)
Supplement: Supplementary file 1 [file Data_Sheet_1.PDF]

Supplementary Material

1 Supplementary Methods

1.1 Comparison of the activity of FAAR and FACR candidates in *S. cerevisiae*

In addition to maFACR, several other FACRs and FAARs have been evaluated. The genes selected are as follows: FACRs from *Acinetobacter baylyi* (AbFACR, NCBI Protein ID: AAC45217.1) and *Acinetobacter* sp. M-1 (AsFACR, NCBI Protein ID: BAB85476.1), and FAARs from *Synechococcus elongatus* PCC 7942 (SeFAAR, NCBI Protein ID: WP_011242364.1), *Anabaena variabilis* ATCC 29413 (AvFAAR, NCBI Protein ID: WP_011319305.1) and *Arthrospira platensis* str. Paraca (ApFAAR, NCBI Protein ID: WP_006617164.1). The genes were codon-optimized for *S. cerevisiae*, synthesized (Genscript, China) and provided as plasmids. The genes were cloned into pYES2/CT (Thermo Fisher, Singapore) as described for construction of pmaFACR. The expression plasmids were transformed into *S. cerevisiae* BY4741 and the enzymes were expressed in 10 mL cultures as described for aldehyde production with pmaFACR (Materials and methods, Section 2.8). To determine the aldehyde production with the FACRs and FAARs, cells were harvested from 5 mL of the cultures. The harvested cells were processed and analyzed by GCMS as described in literature (Foo et al., 2017).

1.2 CRISPR-mediated genome editing

Tools for CRISPR/Cas9-mediated multiplex gene deletion in *S. cerevisiae* were developed by adapting protocols in literature (Jakociunas et al., 2015). Two cloning plasmids were created for constructing guide RNA (gRNA) cassettes and a transcription plasmid was generated for assembly of multiple cassettes for multi-gRNA transcription to achieve multiplex gene deletion in S. cerevisiae. These plasmids employ the multiple cloning site (MCS) of pBluescript II KS(+) (Agilent, Singapore), thus only require common restriction enzymes for constructing the multi-gRNA transcription plasmids. An expression plasmid was constructed to express Cas9.

The plasmids and protocol for the CRISPR/Cas9-mediated gene deletion are shown in Figure S9 and S10. The transcription plasmid pgRNA is a high copy-number plasmid with the MCS of pBluescript II KS(+) and a *URA3* marker with truncated promoter (Foo et al., 2017). The two cloning plasmids, pBS-gRNA1 and pBS-gRNA2, each contain an SNR52p-*Bsa*I-gRNA insertion site-*Bsa*I-structural RNA-Sup4t expression cassette, in which target-specific gRNA sequences created by annealing oligonucleotides can be cloned using the type IIS restriction enzyme *Bsa*I (Figure S9). The gRNA cassettes are cloned within the MCS such that they are flanked by restriction sites. The orientation of the gRNA cassettes in pBS-gRNA1 and pBS-gRNA2 are in opposite directions. Thus, cloning of the gRNA sequences can be distributed equally between the two plasmids and restriction sites can be strategically selected to isolate gRNA cassettes from the cloning plasmids and assembled in pgRNA for transcribing multiple gRNA (Figure S11A). Donor DNAs for gene inactivation were designed as described in literature (Jakociunas et al., 2015) and constructed by annealing complementary oligonucleotides. Multiplex gene deletion was achieved by co-transforming a plasmid expressing Cas9, the pgRNA containing the gRNA cassettes and the respective donor DNAs (Figure S11B).

1.2.1 Plasmid construction

Plasmid pHCas9-L: pHCas9-L was constructed by replacing the *TRP1* marker in p414-TEF1p-Cas9-CYC1t, which was a gift from George Church (Addgene plasmid # 43802) (DiCarlo et al., 2013), with *LEU2*. p414-TEF1p-Cas9-CYC1t was digested with *Xba*I to obtain fragments of 3.7-kb (fragment A) and 5.8-kb (fragment B). Fragment A was dephosphorylated with calf intestine phosphatase. Fragment B was digested with *Dra*III and a 4.8-kb fragment was gel extracted (fragment C). *LEU2* was amplified from pESC-LEU2 (Agilent Technologies) with the primer pair LEU2-F/LEU2-R and digested with *Xba*I/*Dra*III. Fragment A was ligated with equimolar of *LEU2* and fragment C using T4 ligase to obtain pHCas9-L.

*Plasmid pBS-BsaI: Bsa*I restriction site was removed from pBluescript II KS(+) by Quikchange protocol (Agilent) using the primer pairs Amp-BsaI-F/Amp-BsaI-R to generate pBS-BsaI.

Cloning plasmids pBS-gRNA1 and pBS-gRNA2: A duplex DNA fragment containing SNR52p-*Bsa*I-gRNA insertion site-*Bsa*I-structural RNA-Sup4t flanked by *BamH*I/*Pst*I was purchased from Integrated DNA Technologies (Singapore) and cloned into pBS-BsaI to create pBS-gRNA1. The gRNA cassette was amplified from pBS-gRNA1 with the primer pair pgRNA2-F/pgRNA2 (which swapped the *BamH*I/*Pst*I restriction sites) and cloned into pBS-BsaI to created pBS-gRNA2 (Figure S9A).

Transcription plasmid pgRNA: pBluescript II KS(+) was digested with *Sac*I/*Kpn*I and the 100-bp MCS was cloned into the 5.6-kb fragment of pUdGT (Foo et al., 2017) restricted at the same restriction sites to obtain pgRNA.

Cloning of gRNA sequence to create gRNA cassette source plasmids: gRNA sequences were designed using E-CRISP (Heigwer et al., 2014) and complementary oligonucleotides (Table S3) were synthesised with hanging ends for ligation to the *Bsa*I restricted sites on the cloning plasmids. The complementary oligonucleotides (1 µg/µL, 25 µL each) were mixed and annealed by heating at 95ºC for 1 min and cooling at a rate of 0.1 ºC/s to 22ºC on a thermocycler to create the gRNA inserts. To ligate the inserts to the cloning plasmids, 1 µL (1 µg) of gRNA inserts was mixed with 50 ng of pBS-gRNA1 or pBS-gRNA2 (as indicated in Table S4) in a 15 µL reaction containing 1x T4 ligase buffer, 0.1 mg/mL bovine serum albumin, 1 µL *Bsa*I and 1 µL T4 ligase (2 million cohesive end units/mL). The ligation reaction was performed using 25 cycles of 3 min at 37ºC and 4 min at 16ºC ended by 5 min at 50ºC and 5 min at 80ºC (Engler et al., 2009). The ligation mixture was transformed into *E. coli* and purified plasmids were sequenced to ensure successful insertion of the gRNA sequences (Figure S9B).

Assembly of gRNA cassettes in pgRNA for transcription: gRNA cassettes were digested from the gRNA cassette source plasmids and ligated into pgRNA using restriction enzymes as listed in Table S5. To construct the respective plasmids for transcribing gRNAs, 50 ng of digested pgRNA was ligated with equimolar of gRNA cassettes using T4 ligase (Figure S11A).

1.2.2 Gene inactivation in *S. cerevisiae* by CRISPR/Cas9

Competent cells of the respective *S. cerevisiae* parent strains transformed with pHCas9-L were prepared. Donor DNAs were constructed by mixing complementary oligonucleotides (1 µg/µL, 25 µL each) (Table S3) and annealing them by heating at 95ºC for 1 min and cooling at a rate of 0.1 ºC/s to 22ºC on a thermocycler. The desired multi-gRNA-transcribing plasmid was co-transformed with the corresponding donor DNAs into the competent cells and plated on YNB-URA-LEU solid growth

medium. Three colonies were picked for each strain and grown overnight in 5mL YPD. The overnight culture was streaked on YPD solid growth medium to cure the plasmids and genomic DNA was purified from the remaining cells. The mutated sites were PCR amplified using primers listed in Table S3 and sequenced to verify successful disruption of the genes. All selected colonies showed the desired mutations. Single colonies from the YPD plates were spotted on YNB-URA and YNB-LEU single dropout solid growth medium to isolate colonies of the deletion strains cured of both plasmids (Figure S11B).

2 Supplementary Figures

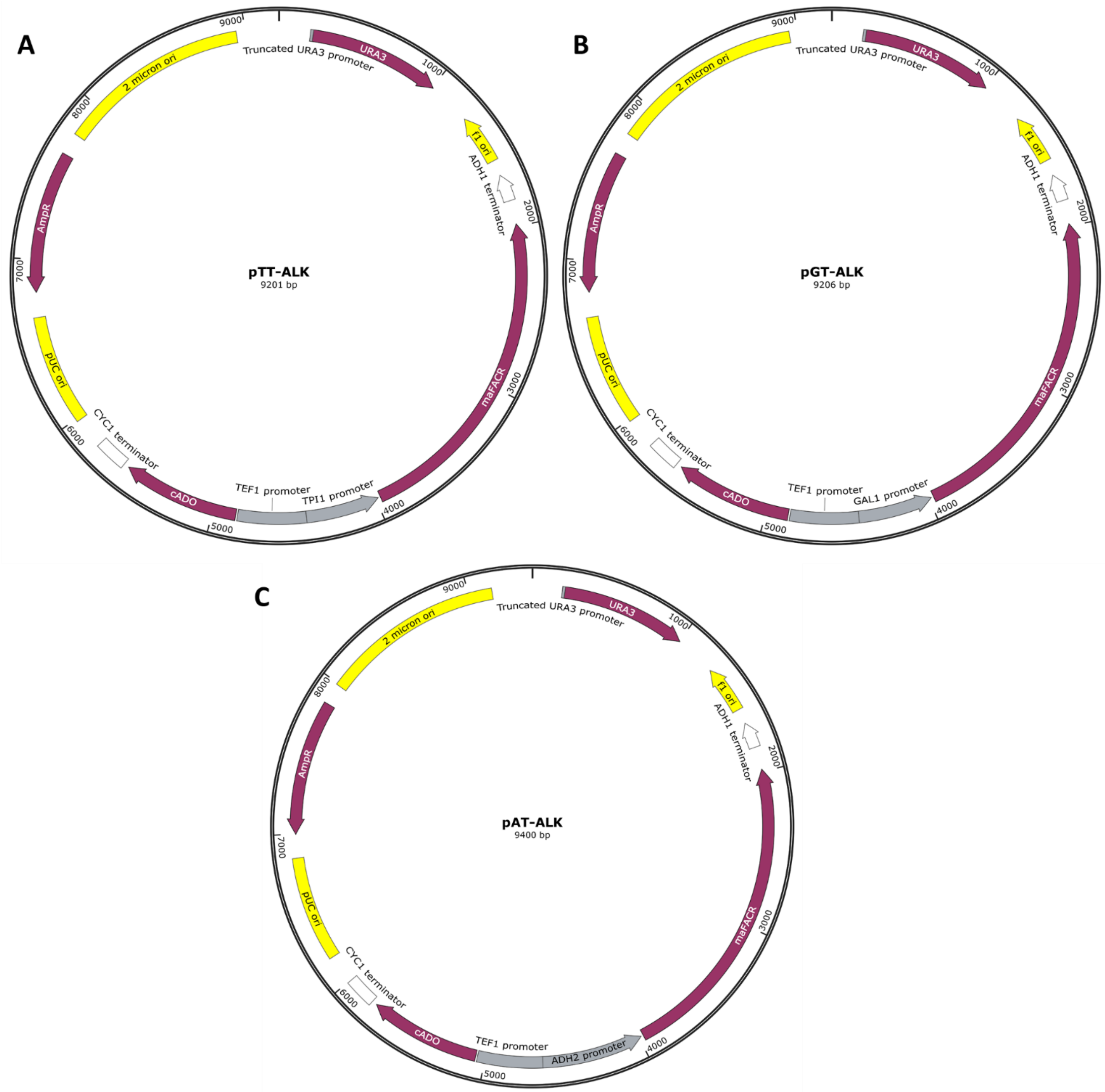

Figure S1. Plasmid maps of constructs used for ALK production. Plasmid maps for **(A)** pTT-ALK, **(B)** pGT-ALK and **(C)** pAT-ALK.

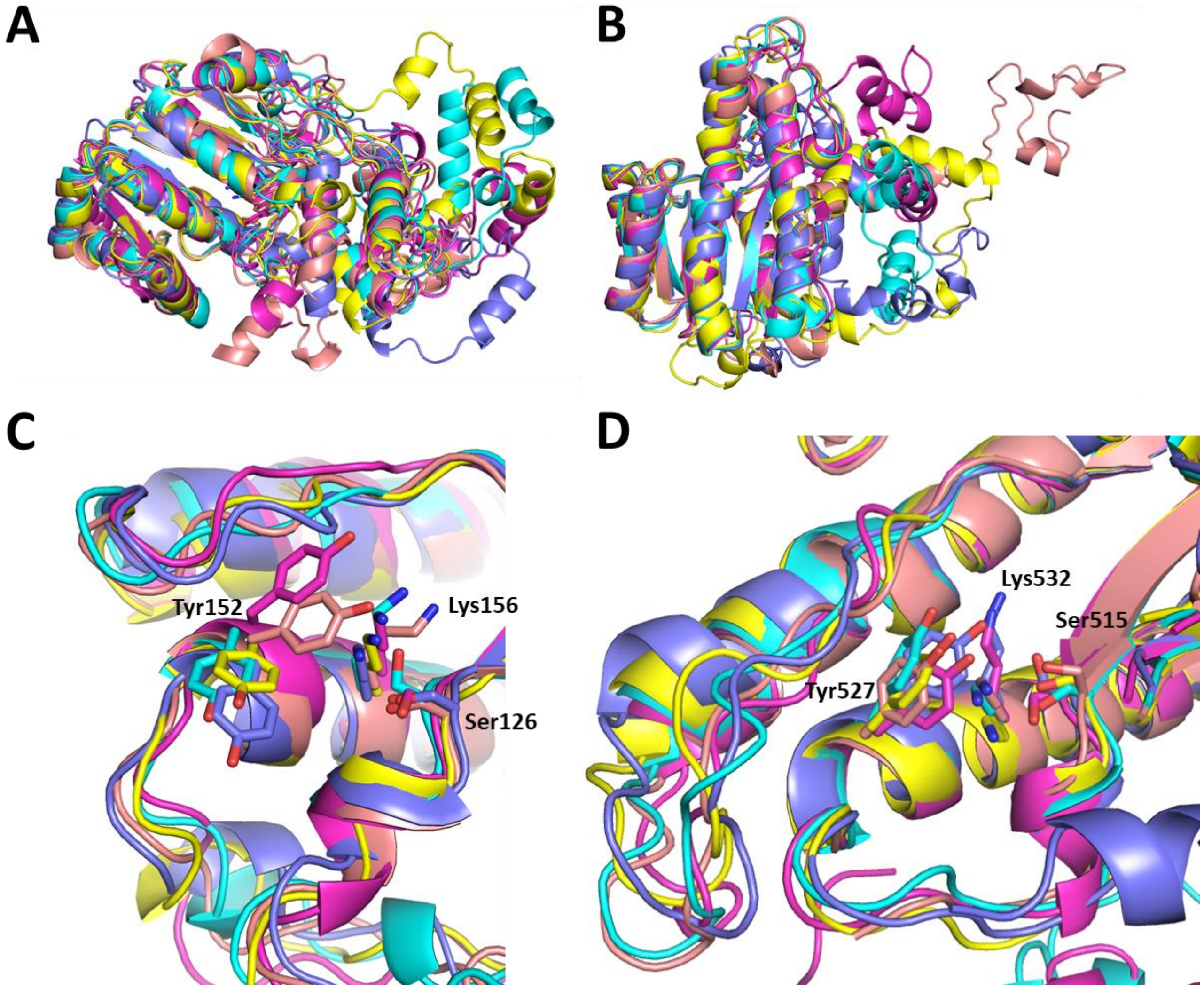

Figure S2. Alignment of predicted structures of maFACR. (A) and **(B)** show the alignment of the N- and C-terminal domains, respectively, from five predicted structures of maFACR. The predicted domains are structurally similar. Relative to the predicted structure in Figure 2, the root-mean-square deviation of atomic positions in the N- and C-terminal domains between the structures range from 0.701-1.152 and 1.454-1.675 Å, respectively. **(C)** and **(D)** illustrate the alignment of the catalytic residues in the N- and C-terminal domains, respectively.

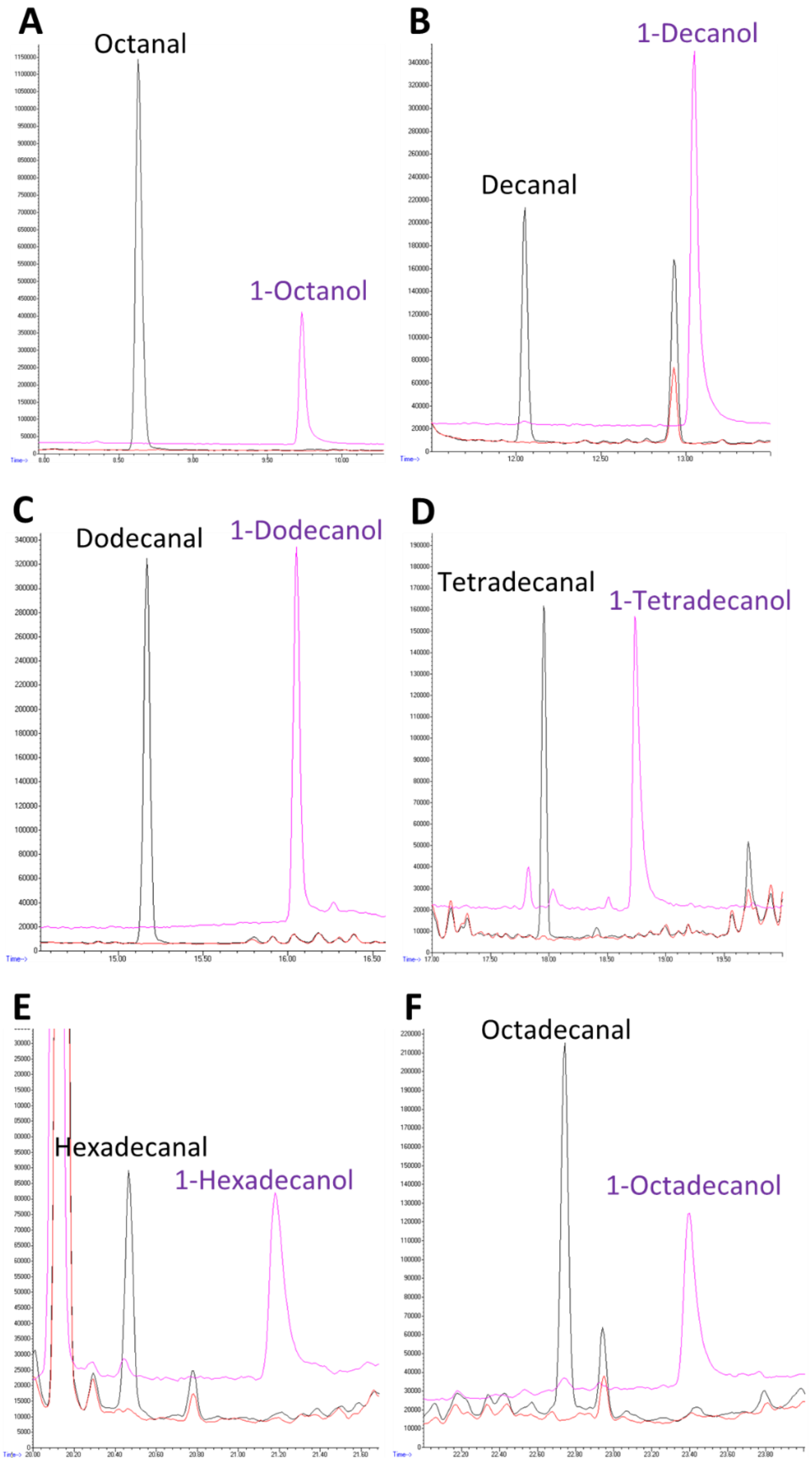

Figure S3. GC-MS analysis of *in vitro* production of fatty aldehydes. Chromatograms of organic extracts from *in vitro* reactions containing fatty acyl-CoAs with and without maFACR$_{SYK}$. The fatty acyl-CoAs used were **(A)** octanoyl-CoA, **(B)** decanoyl-CoA, **(C)** dodecanoyl-CoA, **(D)** tetradecanoyl-CoA, **(E)** hexadecanoyl-CoA and **(F)** octadecanoyl-CoA. In the presence of maFACR$_{SYK}$ (in black), the corresponding aldehydes are observed. No peaks are observed to have the same elution time and mass spectrum as the corresponding authentic fatty alcohol standards (in purple). The controls without maFACR$_{SYK}$ are shown in red.

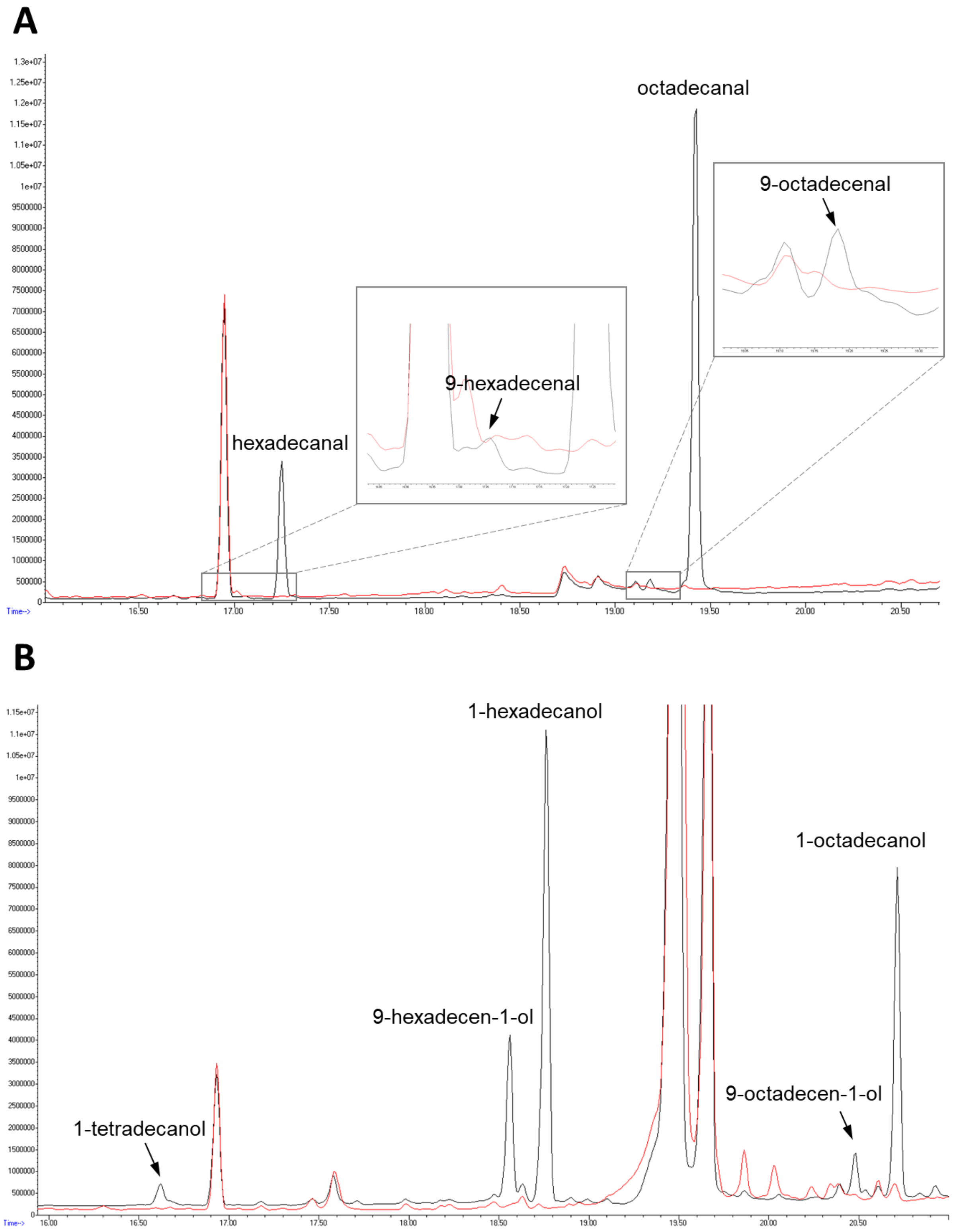

Figure S4. GC-MS analysis of fatty alcohols and fatty aldehydes produced *in vivo* using engineered maFACR. The representative chromatogram shown in black is of the organic extract from *S. cerevisiae* BY4741 expressing maFACRSYK. Peaks corresponding to **(A)** fatty aldehydes and **(B)** alcohols (as trimethylsilyl derivatives) are observed. The control shown in red is from *S. cerevisiae* carrying an empty pYES2/CT vector.

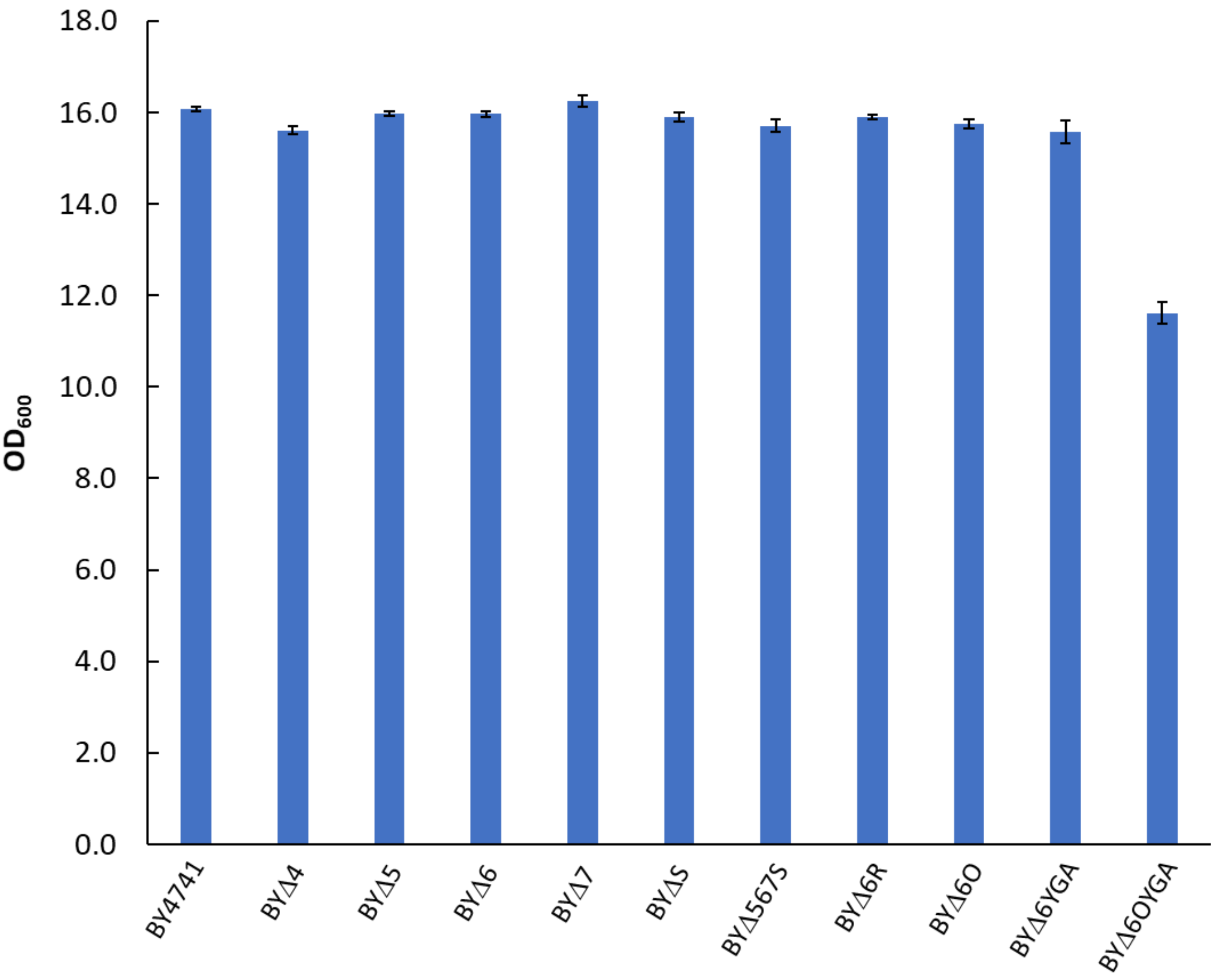

Figure S5. Cell density of various *S. cerevisiae* variants expressing maFACR$_{\mathbf{SYK}}$. The final OD_{600} of the various cultures after 48 h are shown.

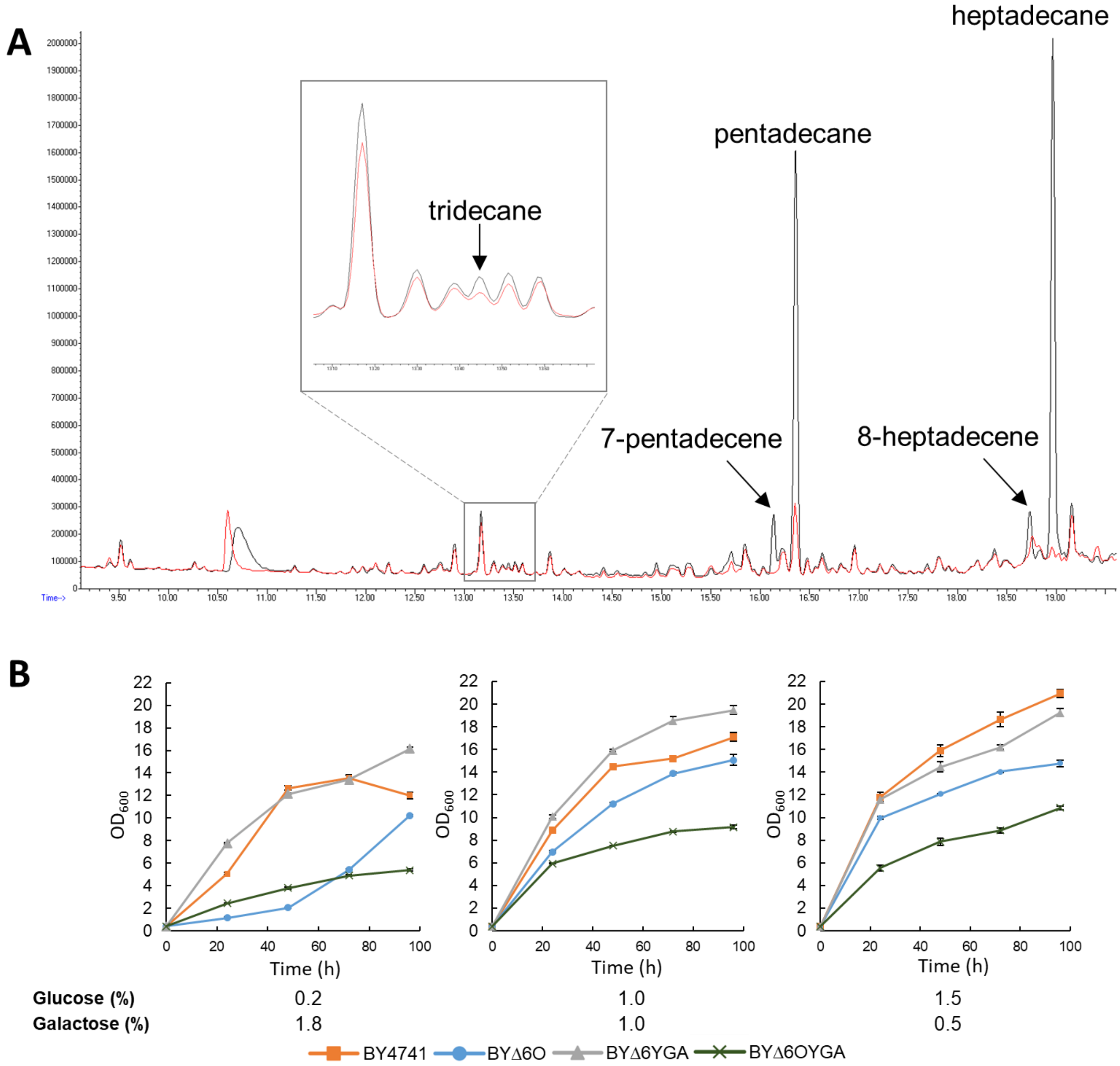

Figure S6. GC-MS analysis and growth profile of various ALK production strains. (A) The chromatogram of organic extract from *S. cerevisiae* BY4741 expressing maFACR$_{SYK}$ and cADO (in black) shows *in vivo* production of ALKs. The control (shown in red) expressed an inactive cADO instead (Foo et al., 2017). **(B)** The growth curves of various *S. cerevisiae* strains harboring pGT-ALK are shown.

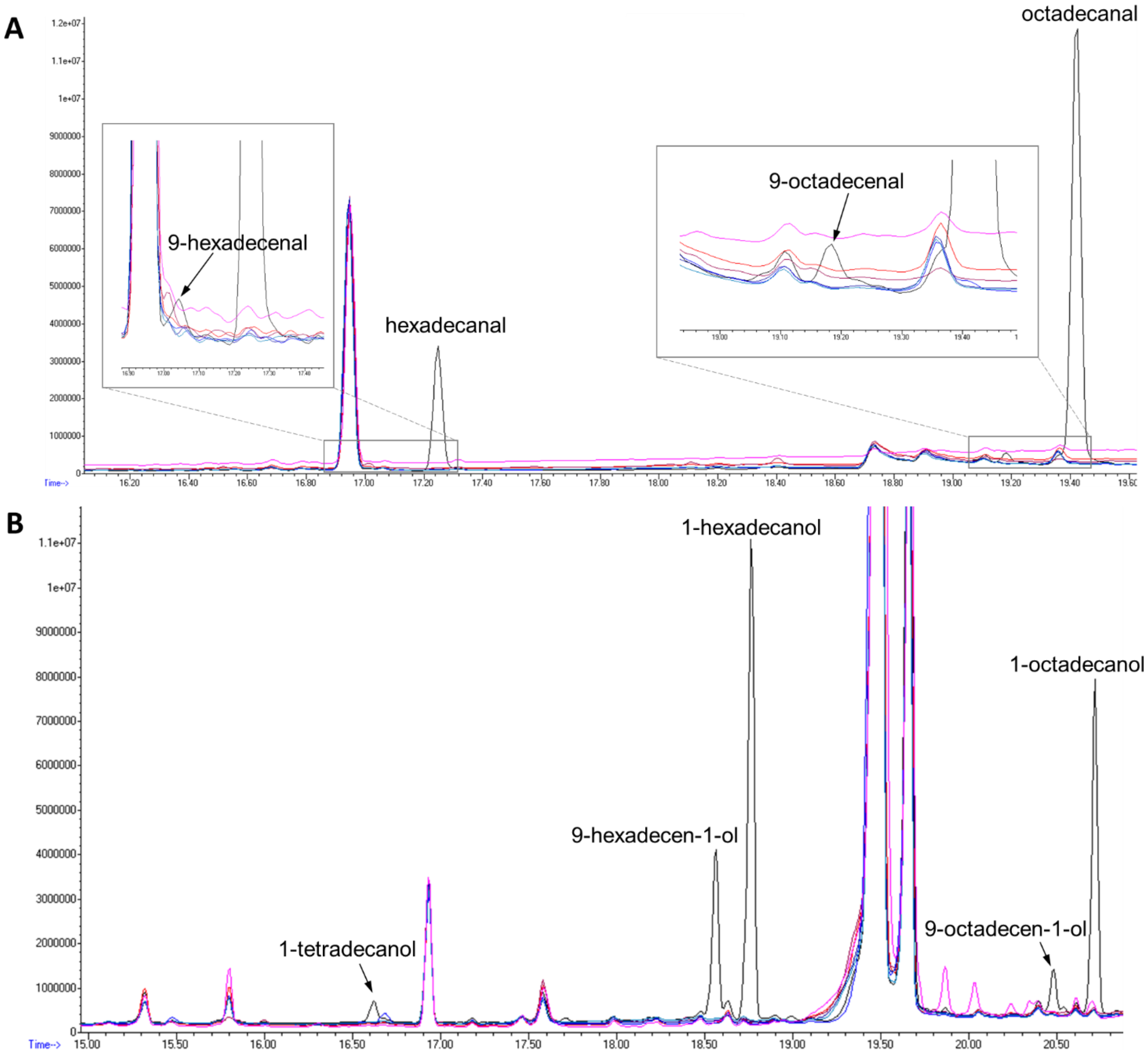

Figure S7. Evaluation of the activities of various FAARs and FACRs. AbFACR (red), AsFACR (maroon), SeFAAR (blue), AvFAAR (dark blue), ApFAAR (green) and maFACR (black) were expressed in *S. cerevisiae*. The control (pink) harbored an empty pYES2/CT vector. Overlay of chromatograms of the organic extracts from the *S. cerevisiae* strains shows that only expression of maFACR$_{SYK}$ produced detectable **(A)** fatty aldehydes and **(B)** alcohols (as trimethylsilyl derivatives).

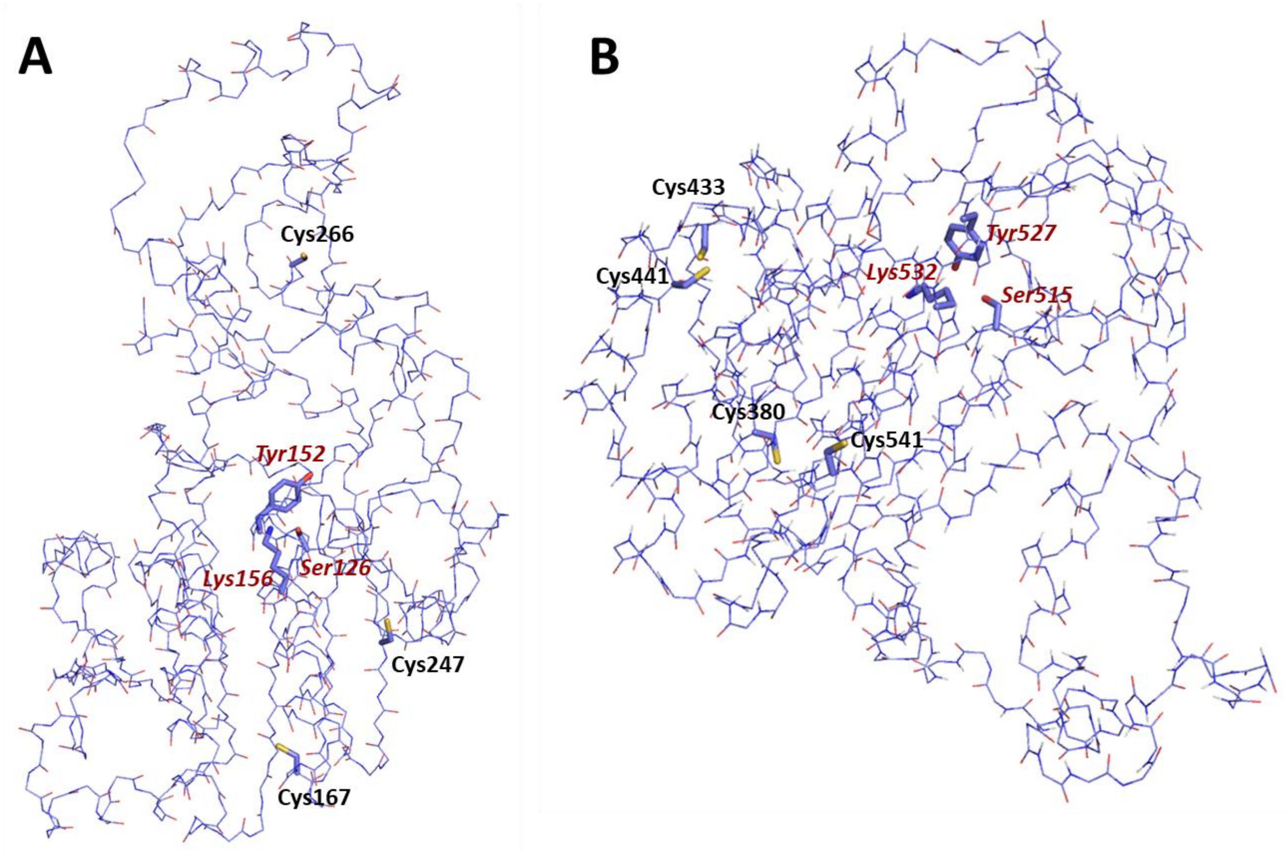

Figure S8. Positions of cysteine residues in maFACR. (A) and **(B)** show the positions of cysteine residues in the N- and C-terminal domains, respectively. The catalytic residues are in red and italicized.

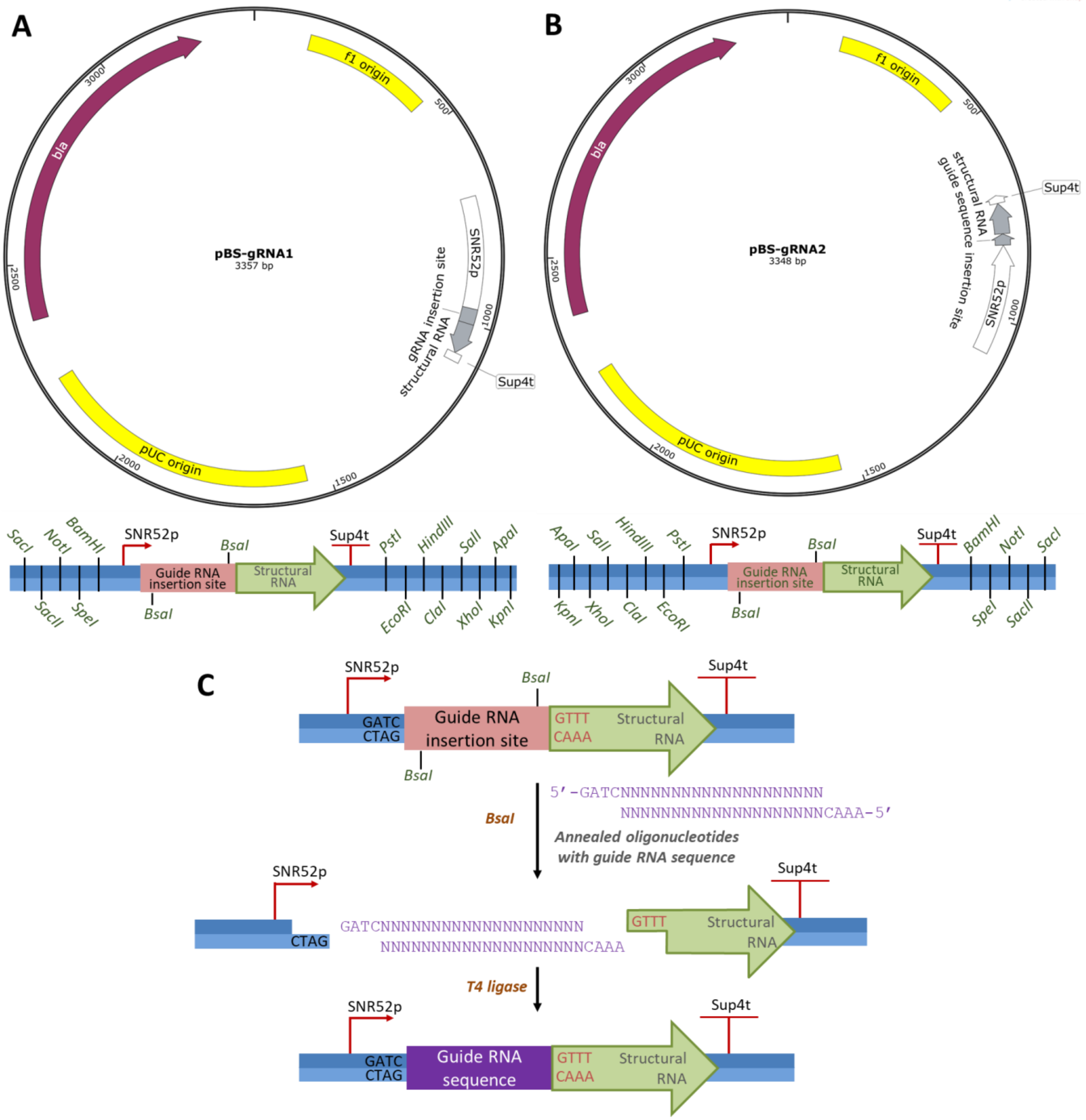

Figure S9. Plasmid maps of constructs used for ALK production. Plasmid maps of the gRNA cloning vectors are shown for **(A)** pBS-gRNA1 and **(B)** pBS-gRNA2. The restriction sites flanking the gRNA cassettes are shown below each cloning vector. **(C)** The procedure for cloning the gRNA sequence into the cloning vectors to create gRNA source plasmids is illustrated.

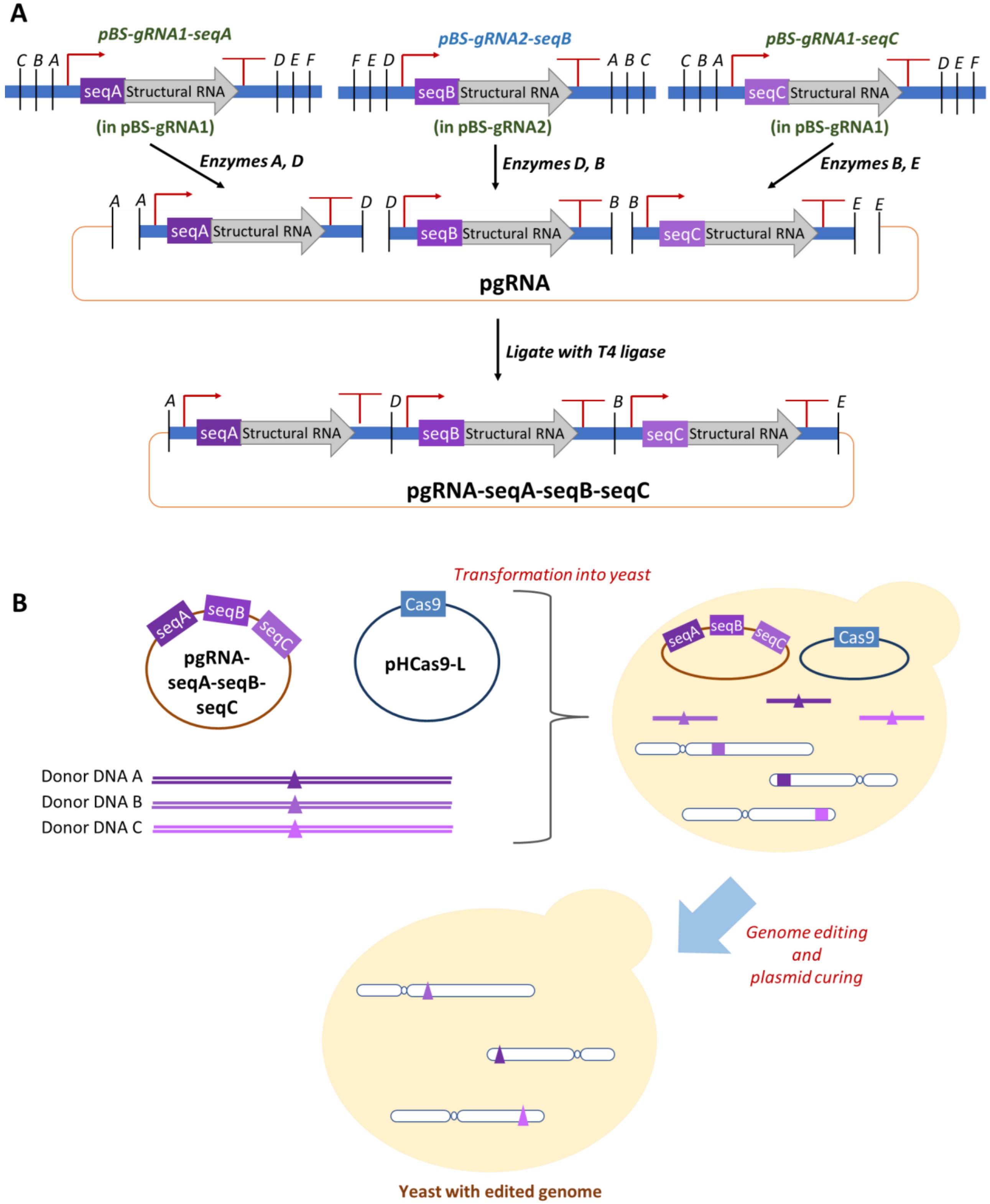

Figure S10. Plasmid maps of constructs used for ALK production. (A) The procedure for assembling multiple gRNA cassettes in pgRNA is illustrated. **(B)** A schematic diagram of the protocol for performing CRISPR/Cas9-mediated gene inactivation in *S. cerevisiae*.

3 Supplementary Tables

Table S1. Plasmids and strains used in this work.

Plasmids or strains	Description	Reference
**Plasmids**		
pMAL-c5x	Ptac-malE, pMB1, AmpR	New England Biolabs
pYES2/CT	P_{GAL1} promoter,2μ origin, AmpR,URA3	Thermo Fisher
pESC-LEU	P_{GAL1}, P_{GAL10} promoters,2μ origin, AmpR,LEU2	Agilent Technologies
p414-TEF1p-Cas9-CYC1t	P_{TEF1}-Cas9-T_{CYC1}, CEN origin, AmpR,*TRP1*	(Jakociunas et al., 2015)
pMAL-maFACR	pMAL-c5x cloned with maFACR	This study
pmaFACR	maFACR cloned into pYES2/CT for expression in *S. cerevisiae*	This study
$pmaFACR_{SYK}$	$maFACR_{SYK}$ cloned into pYES2/CT for expression in *S. cerevisiae*	This study
pUdGT	P_{GAL1}, P_{GAL10} promoters, 2μ ori, URA3, AmpR, truncated P_{URA3}	(Foo et al., 2017)
pUdTT	P_{TPI1}, P_{TEF1} promoters, 2μ ori, URA3, AmpR, truncated P_{URA3}	This study
pUdAT	P_{ADH2}, P_{TEF1} promoters, 2μ ori, URA3, AmpR, truncated P_{URA3}	This study
pUdGT-cADO	pUdGT with cADO under P_{TEF1}	This study
pUdTT-cADO	pUdTT with cADO under P_{TEF1}	This study
pUdAT-cADO	pUdAT with cADO under P_{TEF1}	This study
pGT-ALK	pUdGT-cADO with $maFACR_{SYK}$ under P_{GAL1}	This study
pTT-ALK	pUdTT-cADO with $maFACR_{SYK}$ under P_{TPI1}	This study
pAT-ALK	pUdAT-cADO with $maFACR_{SYK}$ under P_{ADH2}	This study
**Strains**		
BY4741	MATa *his3Δ1 leu2Δ0 met15Δ0 ura3Δ0*	ATCC
BYΔ1	MATa *his3Δ1 leu2Δ0 met15Δ0 ura3Δ0 adh1Δ0*	This study
BYΔ2	MATa *his3Δ1 leu2Δ0 met15Δ0 ura3Δ0 adh2Δ0*	This study
BYΔ3	MATa *his3Δ1 leu2Δ0 met15Δ0 ura3Δ0 adh3Δ0*	This study
BYΔ4	MATa *his3Δ1 leu2Δ0 met15Δ0 ura3Δ0 adh4Δ0*	This study
BYΔ5	*MATa his3Δ1 leu2Δ0 met15Δ0 ura3Δ0 adh5Δ0*	This study

BYΔ6	*MATa his3Δ1 leu2Δ0 met15Δ0 ura3Δ0 adh6Δ0*	This study
BYΔ7	MATa *his3Δ1 leu2Δ0 met15Δ0 ura3Δ0 adh7Δ0*	This study
BYΔS	MATa *his3Δ1 leu2Δ0 met15Δ0 ura3Δ0 sfa1Δ0*	This study
BYΔ567S	MATa *his3Δ1 leu2Δ0 met15Δ0 ura3Δ0 adh5Δ adh6Δ0 adh7Δ sfa1Δ*	This study
BYΔ6YGA	MATa *his3Δ1 leu2Δ0 met15Δ0 ura3Δ0 adh6Δ0 YDR541CΔ gre2Δ ari1Δ*	This study
BYΔ6H	MATa *his3Δ1 leu2Δ0 met15Δ0 ura3Δ0 adh6Δ0 hfd1Δ*	This study
BYΔ6R	MATa *his3Δ1 leu2Δ0 met15Δ0 ura3Δ0 adh6Δ0 rpd3Δ*	This study
BYΔ6O	MATa *his3Δ1 leu2Δ0 met15Δ0 ura3Δ0 adh6Δ0 opi1Δ*	This study
BYΔ6OYGA	MATa *his3Δ1 leu2Δ0 met15Δ0 ura3Δ0 adh6Δ0 opi1Δ YDR541CΔ gre2Δ ari1Δ*	This study
BY4741/pmaFACR	BY4741 with pmaFACR	This study
BY4741/pmaFACR$_{SYK}$	BY4741 with pmaFACR$_{SYK}$	This study
BYΔ1/pmaFACR$_{SYK}$	BYΔ1 with pmaFACR$_{SYK}$	This study
BYΔ2/pmaFACR$_{SYK}$	BYΔ2 with pmaFACR$_{SYK}$	This study
BYΔ3/pmaFACR$_{SYK}$	BYΔ3 with pmaFACR$_{SYK}$	This study
BYΔ4/pmaFACR$_{SYK}$	BYΔ4 with pmaFACR$_{SYK}$	This study
BYΔ5/pmaFACR$_{SYK}$	BYΔ5 with pmaFACR$_{SYK}$	This study
BYΔ6/pmaFACR$_{SYK}$	BYΔ6 with pmaFACR$_{SYK}$	This study
BYΔ7/pmaFACR$_{SYK}$	BYΔ7 with pmaFACR$_{SYK}$	This study
BYΔS/pmaFACR$_{SYK}$	BYΔS with pmaFACR$_{SYK}$	This study
BYΔ567S/pmaFACR$_{SYK}$	BYΔ567S with pmaFACR$_{SYK}$	This study
BYΔ6YGA/pmaFACR$_{SYK}$	BYΔ6YGA with pmaFACR$_{SYK}$	This study
BYΔ6H/pmaFACR$_{SYK}$	BYΔ6H with pmaFACR$_{SYK}$	This study
BYΔ6R/pmaFACR$_{SYK}$	BYΔ6R with pmaFACR$_{SYK}$	This study
BYΔ6O/pmaFACR$_{SYK}$	BYΔ6O with pmaFACR$_{SYK}$	This study
BYΔ6OYGA/pmaFACR$_{SYK}$	BYΔ6OYGA with pmaFACR$_{SYK}$	This study
BY4741/pTT-ALK	BY4741 with pTT-ALK	This study
BY4741/pGT-ALK	BY4741 with pGT-ALK	This study
BYΔ6O/pGT-ALK	BYΔ6O with pGT-ALK	This study
BYΔ6YGA/pGT-ALK	BYΔ6YGA with pGT-ALK	This study
BYΔ6OYGA/pGT-ALK	BYΔ6OYGA with pGT-ALK	This study
BYΔ6OYGA/pAT-ALK	BYΔ6OYGA with pAT-ALK	This study

Table S2. Oligonucleotides used in this work. Restriction sites are underlined.

Name	Sequence
pESC-pmt-TEF1-F	CGCTCGGCGGCTTCTAATCCGTACTTTTCATAGCTTCAAAATGTTTCTAC
pESC-pmt-TEF1-R	CGGGCCCTATAGTGAGTCGTATTAC
pESC-pmt-ADH2-F	GTAGAAACATTTTGAAGCTATGAAATGTTTGTTTGAAGAGACTAATCAAA
pESC-pmt-ADH2-R	AGGGTTGAATTCGAATTGTGTATTACGATATAGTTAATAGT
pESC-pmt-TPI1-F	GTAGAAACATTTTGAAGCTATGAAAGTGATCCTACTTATTCCCTTCGAGATTATAT
pESC-pmt-TPI1-R	AGGGTTGAATTCGAATTTTTAGTTTATGTATGTGTTTTTTGTAGT
S126A-F	CCATCACGTTTCTGCTATCGCCGCTGCAG
S126A-R	CTGCAGCGGCGATAGCAGAAACGTGATGG
Y152F-F	AAATTGGATCATCCATTTTTGAGAACTAAGCACG
Y152F-R	CGTGCTTAGTTCTCAAAAATGGATGATCCAATTT
K156A-F	CCATATTTGAGAACTGCTCACGAAAGTGAAAAA
K156A-R	TTTTTCACTTTCGTGAGCAGTTCTCAAATATGG
S515A-F	GTCAATATATCCGCTATCGGTGTATTAACAAAC
S515A-R	GTTTGTTAATACACCGATAGCGGATATATTGAC
Y528F-F	CCTAGATTCTCAGCATTTGTTTCTTCAAAATCAGC
Y528F-R	GCTGATTTTGAAGAAACAAATGCTGAGAATCTAGG
K532A-F	GCATACGTTTCTTCAGCTTCAGCTTTGGACGCA
K532A-R	TGCGTCCAAAGCTGAAGCTGAAGAAACGTATGC
Nter-F	AATACGACTCACTATAGGGAATATT
Nter-R	GCCAGTCTCGAGACAAACTTTACCCTCTACGGTACC
Cter-F	GCCAGTAAGCTTAAAAATGATAGAAGTCCCAAGATTACCTTCAT
Cter-R	GCTTACCTTCGAAGGGCCCTCTAGA
maFACR-MAL-F	TTCTATATTAATAACTACTTCTTGACTGGTGGTACTG
maFACR-MAL-R	ATATTTGAATTCTTACCAATAGATACCTCTCATAATGGCT
Cter-MAL-F	TTCTATATTAATATAGAAGTCCCAAGATTACCTTCAT
Nter-MAL-R	ATATTTGAATTCTTAACAAACTTTACCCTCTACGGTACC
pESC-maFACR-F	ATATTTGAATTCAAAAATGAACTACTTCTTGACTGGT
pESC-maFACR-R	TTCTATGAGCTCTCACCAATAGATACCTCTCATAATGGCT
Amp-BsaI-F	TGCAATGATACCGCGTGACCCACGCTCACCGGC

Amp-BsaI-R	GCCGGTGAGCGTGGGTCACGCGGTATCATTGCA
pgRNA2-F	TAATGACTGCAGTCTTTGAAAAGATAATGTATGATTA
pgRNA2-R	TGTAGAGGATCCAGACATAAAAAACAAAAAAAGCACC
LEU2-F	ATATTTCACGTAGTGTTTTTTTTCCAATAGGTGGTTAGCAATCGTC
LEU2-R	GCTTTATCTAGAGTCGTTAAGGCCGTTTCTGACAGAG

Table S3. Oligonucleotides used in this work for CRISPR/Cas9.

Oligonucleotides	Sequences	Targets
gRNA		
gRNA-ADH5-F	GATCTGCTGGTGTTGTTGTTAAGT	*ADH5*
gRNA-ADH5-R	AAACACTTAACAACAACACCAGCA	*ADH5*
gRNA-ADH7-F	GATCTGGATCCAAGTGCCACACTG	*ADH7*
gRNA-ADH7-R	AAACCAGTGTGGCACTTGGATCCA	*ADH7*
gRNA-SFA1-F	GATCCCTTGCGTTCTGGGCCACGA	*SFA1*
gRNA-SFA1-R	AAACTCGTGGCCCAGAACGCAAGG	*SFA1*
gRNA-HFD1-F	GATCGAAGAATCAGATACTCTCCG	*HFD1*
gRNA-HFD1-R	AAACCGGAGAGTATCTGATTCTTC	*HFD1*
gRNA-YDR541C-F	GATCGCATTTGGATGAGAAATGTC	*YDR541C*
gRNA-YDR541C-R	AAACGACATTTCTCATCCAAATGC	*YDR541C*
gRNA-GRE2-F	GATCCCTGTTGAAGGAAGACTATA	*GRE2*
gRNA-GRE2-R	AAACTATAGTCTTCCTTCAACAGG	*GRE2*
gRNA-ARI1-F	GATCGTTTTCGTTTCTGGCGCAAC	*ARI1*
gRNA-ARI1-R	AAACGTTGCGCCAGAAACGAAAAC	*ARI1*
gRNA-RPD3-F	GATCGCAACGCGTCTTTTATCGCT	*RPD3*
gRNA-RPD3-R	AAACAGCGATAAAAGACGCGTTGC	*RPD3*
gRNA-OPI1-F	GATCTTTAGGATTATCAGAGGAAG	*OPI1*
gRNA-OPI1-R	AAACCTTCCTCTGATAATCCTAAA	*OPI1*
Donor DNA		
dnrDNA-ADH5-F	AATTTCCATTAATCGGTGGTCACGAAGGTGCTGGTGTTGTTGTTAAGTTAAATCTAACGTTAAG GGCTGGAAAGTCGGTGATTTTGCAGG	*ADH5*
dnrDNA-ADH5-R	CCTGCAAAATCACCGACTTTCCAGCCCTTAACGTTAGATTTAACTTAACAACAACACCAGCACC TTCGTGACCACCGATTAATGGAAATT	*ADH5*

dnrDNA-ADH7-F	ATGAAATAATTGGCCGCGTGGTGAAGGTTGGATCCAAGTGCCACACTGTAAAAAAATCGGTGAC CGTGTTGGTGTTGGTGCCCAAGCCTT	*ADH7*
dnrDNA-ADH7-R	AAGGCTTGGGCACCAACACCAACACGGTCACCGATTTTTTTACAGTGTGGCACTTGGATCCAAC CTTCACCACGCGGCCAATTATTTCAT	*ADH7*
dnrDNA-SFA1-F	ATCAGGCTCTGATCCAGAAGGACTTTTCCCTTGCGTTCTGGGCCACGATAAGCCGGTATCGTAG AATCTGTAGGCGATGATGTCATAACA	*SFA1*
dnrDNA-SFA1-R	TGTTATGACATCATCGCCTACAGATTCTACGATACCGGCTTATCGTGGCCCAGAACGCAAGGGA AAAGTCCTTCTGGATCAGAGCCTGAT	*SFA1*
dnrDNA-HFD1-F	TATACTTCACCTAATTGAGATTTTACCAAAATTGATCAATAACGGAGAGTATCTGATTCTTCTC CTCCATTTATGTTTGGTAAAACAATC	*HFD1*
dnrDNA-HFD1-R	GATTGTTTTACCAAACATAAATGGAGGAGAAGAATCAGATACTCTCCGTTATTGATCAATTTTG GTAAAATCTCAATTAGGTGAAGTATA	*HFD1*
dnrDNA-YDR541C-F	ACAATTTCAACATAACCCTAATTTAACTTTAGAAATTGTTAAGACATTTCTCATCCAAATGCTT TCGATAAGGTTCTGCAGAAACGTGGA	*YDR541C*
dnrDNA-YDR541C-R	TCCACGTTTCTGCAGAACCTTATCGAAAGCATTTGGATGAGAAATGTCTTAACAATTTCTAAAG TTAAATTAGGGTTATGTTGAAATTGT	*YDR541C*
dnrDNA-GRE2-F	GGTTCATTGCCCAACACATTGTCGATCTCCTGTTGAAGGAAGACTATATAACATCGGTTCTGCC AGAAGTCAAGAAAAGGCCGAGAATTT	*GRE2*
dnrDNA-GRE2-R	AAATTCTCGGCCTTTTCTTGACTTCTGGCAGAACCGATGTTATATAGTCTTCCTTCAACAGGAG ATCGACAATGTGTTGGGCAATGAACC	*GRE2*
dnrDNA-ARI1-F	AGTAATAATTATGACTACTGATACCACTGTTTTCGTTTCTGGCGCAACTAATTCATTGCTCTAC ACATTATGAACGATCTGTTGAAAGCT	*ARI1*
dnrDNA-ARI1-R	AGCTTTCAACAGATCGTTCATAATGTGTAGAGCAATGAATTAGTTGCGCCAGAAACGAAAACAG TGGTATCAGTAGTCATAATTATTACT	*ARI1*
dnrDNA-RPD3-F	GGTATATGAAGCAACACCTTTTGATCCGATCACGGTCAATAAAGCGATAAAAGACGCGTTGCAT ATTTTTACGATGCAGACGTTGGGAAC	*RPD3*
dnrDNA-RPD3-R	GTTCCCAACGTCTGCATCGTAAAAATATGCAACGCGTCTTTTATCGCTTTATTGACCGTGATCG GATCAAAAGGTGTTGCTTCATATACC	*RPD3*

dnrDNA-OPI1-F	ACTAGTCATTGATGTCTGAAAATCAACGTTTAGGATTATCAGAGGAAGTAAAGAAGCGGCTGAA GTACTTGGGGTGTTGAAACAATCATG	*OPI1*
dnrDNA-OPI1-R	CATGATTGTTTCAACACCCCAAGTACTTCAGCCGCTTCTTTACTTCCTCTGATAATCCTAAACG TTGATTTTCAGACATCAATGACTAGT	*OPI1*
Verification		
ADH5-F	ATGCCTTCGCAAGTCATTCCTGAAA	*ADH5*
ADH5-R	TCATTTAGAAGTCTCAACAACATAT	*ADH5*
ADH7-F	ATGCTTTACCCAGAAAAATTTCAGG	*ADH7*
ADH7-R	CTATTTATGGAATTTCTTATCATAA	*ADH7*
SFA1-F	ATGTCCGCCGCTACTGTTGGTAAAC	*SFA1*
SFA1-R	CTATTTTATTTCATCAGACTTCAAG	*SFA1*
HFD1-F	TCATGAGGAAGAACTGATCGATGCT	*HFD1*
HFD1-R	GCTGCTGCCAATGGGGCAAATGCTA	*HFD1*
YDR541C-F	GTAAAGTGTGTAAAACGACAGAATC	*YDR541C*
YDR541C-R	TTTTGTACCTTCTAACGCGGGAATC	*YDR541C*
GRE2-F	GCGTGAACTATGTCATATTTGCGAT	*GRE2*
GRE2-R	AACTATCTTGATATCCTTGCCGTGC	*GRE2*
ARI1-F	ACGCCAGCTCTCTAGTTACAGTTTC	*ARI1*
ARI1-R	CAATATCTTCCACAATTTCCATCGA	*ARI1*
RPD3-F	TTGAAAGCACAAGAGCAGCTCAC	*RPD3*
RPD3-R	AAGCCATAATTCATAATAAGGGAAT	*RPD3*
OPI1-F	AGGCTGGAAGAACTATATAGACAAG	*OPI1*
OPI1-R	AGAATGTCACTACGTTACTGATAAT	*OPI1*

Table S4. Source plasmids created to contain gRNA cassettes for CRISPR/Cas9.

Source plasmids	Cloning vectors used	Remarks
pBS-gRNA-ADH5	pBS-gRNA1	Contains gRNA cassette for ADH5 disruption
pBS-gRNA-ADH7	pBS-gRNA2	Contains gRNA cassette for AD7 disruption
pBS-gRNA-SFA1	pBS-gRNA1	Contains gRNA cassette for SFA1 disruption
pBS-gRNA-OPI1	pBS-gRNA1	Contains gRNA cassette for OPI1 disruption
pBS-gRNA-RPD3	pBS-gRNA1	Contains gRNA cassette for RPD3 disruption
pBS-gRNA-YDR541C	pBS-gRNA1	Contains gRNA cassette for YDR541C disruption
pBS-gRNA-GRE2	pBS-gRNA2	Contains gRNA cassette for GRE2 disruption
pBS-gRNA-ARI1	pBS-gRNA1	Contains gRNA cassette for ARI1 disruption
pBS-gRNA-HFD1	pBS-gRNA1	Contains gRNA cassette for HFD1 disruption

Table S5. Plasmids created to transcribe gRNA for CRISPR/Cas9.

Plasmids	Source plasmids used	Restriction sites used	Remarks
pgRNA-57S	pBS-gRNA-ADH5<br>pBS-gRNA-ADH7<br>pBS-gRNA-SFA1	*BamH*I, *Pst*I<br>*Pst*I, *Spe*I<br>*Spe*I, *EcoR*I	Plasmid for transcribing gRNA for combinatorial disruption of *ADH5*, *ADH7* and *SFA1*. Cloned into pgRNA digested with *BamH*I and *EcoR*I
pgRNA-YGA	pBS-gRNA-YDR541C<br>pBS-gRNA-GRE2<br>pBS-gRNA-ARI1	*BamH*I, *Pst*I<br>*Pst*I, *Spe*I<br>*Spe*I, *EcoR*I	Plasmid for transcribing gRNA for combinatorial disruption of *YDR541C*, *GRE2* and *ARI1*. Cloned into pgRNA digested with *BamH*I and *EcoR*I
pgRNA-OPI1	pBS-gRNA-OPI1	*BamH*I, *Pst*I	Plasmid for transcribing gRNA for disrupting *OPI1*. Cloned into pgRNA digested with *BamH*I and *Pst*I
pgRNA-RPD3	pBS-gRNA-RPD3	*BamH*I, *Pst*I	Plasmid for transcribing gRNA for disrupting *RPD3*. Cloned into pgRNA digested with *BamH*I and *Pst*I
pgRNA-HFD1	pBS-gRNA-HFD1	*BamH*I, *Pst*I	Plasmid for transcribing gRNA for disrupting *HFD1*. Cloned into pgRNA digested with *BamH*I and *Pst*I

Table S6. Codon-optimized gene sequences of enzymes

Gene	Sequence
maFACR	ATGAACTACTTCTTGACTGGTGGTACTGGTTTTATTGGTAGATTCTTGGTTGAAAAATTGTTGGCTAGAGGTGGTACTGTTTATGTATTAGTTAGAGAACAATC<br>TCAGGATAAGTTGGAAAGATTGAGAGAAAGATGGGGTGCCGATGACAAACAAGTCAAGGCTGTAATAGGTGACTTGACATCTAAAAATTTGGGTATCGATGCTA<br>AGACCTTGAAGTCTTTAAAGGGTAACATCGATCATGTATTCCACTTAGCTGCAGTTTATGATATGGGTGCCGACGAAGAAGCTCAAGCCGCTACTAATATTGAA<br>GGTACAAGAGCAGCCGTCCAAGCTGCAGAAGCCATGGGTGCTAAACATTTCCATCACGTTTCTGATATCGCCGCTGCAGGTTTGTTCAAGGGTATTTTTAGAGA<br>AGACATGTTTGAAGAAGCTGAAAAATTGGATCATCCATTTTTGAGAACTGCTCACGAAAGTGAAAAAGTTGTCAGAGAAGAGTGTAAGGTACCATTCAGAATCT<br>ACAGACCTGGTATGGTTATTGGTCATTCTGAAACCGGTGAAATGGATAAAGTTGACGGTCCATACTACTTTTTCAAGATGATCCAAAAGATTAGACACGCTTTG<br>CCACAATGGGTTCCTACTATCGGTATTGAAGGTGGTAGATTAAACATCGTACCTGTTGATTTTGTAGTTGATGCATTGGACCATATTGCCCACTTAGAAGGTGA<br>AGATGGTAATTGTTTCCATTTGGTCGATTCTGACCCATACAAAGTAGGTGAAATCTTGAACATATTTTGCGAAGCAGGTCACGCCCCTAGAATGGGTATGAGAA<br>TCGATTCAAGAATGTTCGGTTTCATTCCACCTTTTATAAGACAATCTATTAAAAATTTGCCACCAGTTAAGAGAATTACTGGTGCTTTGTTAGATGACATGGGT<br>ATTCCACCTTCTGTTATGTCATTCATAAACTACCCAACCAGATTTGACACTAGAGAATTGGAAAGAGTTTTGAAGGGTACAGATATAGAAGTCCCAAGATTACC<br>TTCATATGCACCAGTTATATGGGATTACTGGGAAAGAAATTTGGATCCTGATTTGTTTAAAGATAGAACATTGAAGGGTACCGTAGAGGGTAAAGTTTGTGTCG<br>TAACAGGTGCTACCTCCGGTATTGGTTTGGCTACAGCAGAAAAATTGGCCGAAGCTGGTGCAATCTTGGTTATTGGTGCAAGAACTAAGGAAACATTGGATGAA<br>GTTGCCGCTAGTTTAGAAGCAAAAGGTGGTAATGTCCATGCCTATCAATGTGATTTCTCTGACATGGATGACTGCGATAGATTTGTTAAAACTGTCTTGGATAA<br>CCATGGTCACGTCGACGTATTAGTTAATAACGCTGGTAGATCCATAAGAAGAAGTTTGGCATTATCTTTTGATAGATTCCATGACTTCGAAAGAACAATGCAAT<br>TGAACTACTTCGGTTCAGTTAGATTGATTATGGGTTTTGCCCCAGCTATGTTGGAAAGAAGAAGAGGTCATGTTGTCAATATATCCAGTATCGGTGTATTAACA<br>AACGCTCCTAGATTCTCAGCATACGTTTCTTCAAAATCAGCTTTGGACGCATTTTCCAGATGCGCAGCCGCTGAATGGTCCGATAGAAACGTCACCTTTACTAC<br>AATTAACATGCCATTGGTAAAGACCCCAATGATTGCTCCTACTAAAATCTATGATTCTGTTCCAACCTTGACTCCTGACGAAGCAGCCCAAATGGTTGCAGATG<br>CCATAGTCTACAGACCAAAGAGAATCGCTACTAGATTGGGTGTCTTCGCACAAGTATTGCATGCTTTGGCACCTAAGATGGGTGAAATCATCATGAACACAGGT<br>TACAGAATGTTTCCAGATTCACCTGCTGCAGCCGGTTCCAAAAGTGGTGAAAAGCCTAAAGTTTCAACAGAACAAGTCGCATTCGCAGCCATTATGAGAGGTAT<br>CTATTGGTGA
cADO	ATGCCTCAATTGGAAGCTTCTTTGGAATTGGATTTCCAATCCGAATCCTACAAGGATGCTTACTCTAGAATCAACGCCATCGTTATTGAAGGTGAACAAGAAGC<br>TTTCGACAACTACAATAGATTGGCTGAAATGTTGCCAGACCAAAGAGATGAATTGCATAAGTTGGCCAAGATGGAACAAAGACATATGAAGGGTTTCATGGCTT<br>GTGGTAAGAACTTGTCTGTTACTCCAGATATGGGTTTCGCTCAAAAGTTCTTTGAAAGATTGCACGAAAACTTCAAAGCTGCTGCTGCTGAAGGTAAAGTTGTT<br>ACTTGCTTGTTGATCCAATCCTTGATCATTGAATGCTTTGCTATTGCTGCCTACAACATCTATATTCCAGTTGCTGATGCTTTCGCCAGAAAAATTACTGAAGG<br>TGTTGTCAGAGATGAATACTTGCATAGAAACTTCGGTGAAGAATGGTTGAAGGCTAATTTCGATGCTTCTAAGGCCGAATTGGAAGAAGCTAATAGACAAAACT<br>TGCCATTGGTCTGGTTGATGTTGAATGAAGTTGCAGATGATGCCAGAGAATTGGGTATGGAAAGAGAATCTTTGGTCGAAGATTTCATGATTGCTTACGGTGAA<br>GCCTTGGAAAACATTGGTTTCACTACCAGAGAAATCATGAGAATGTCTGCTTATGGTTTGGCTGCTGTTCACCATCACCATCACCATTAA
AbFACR	ATGAACAAAAAGTTGGAAGCCTTGTTCAGAGAAAACGTTAAGGGTAAGGTTGCTTTGATTACTGGTGCTTCTTCTGGTATTGGTTTGACTATTGCTAAGAGAAT<br>TGCTGCTGCTGGTGCTCATGTTTTGTTGGTTGCTAGAACTCAAGAAACCTTGGAAGAAGTTAAGGCTGCCATTGAACAACAAGGTGGTCAAGCTAGTATTTTCC<br>CATGTGATTTGACCGATATGAACGCCATTGATCAATTGTCCCAACAAATTATGGCCTCCGTTGATCATGTCGATTTCTTGATTAACAACGCCGGTAGATCCATT<br>AGAAGAGCTGTTCATGAATCCTTCGATAGATTCCACGATTTCGAAAGAACCATGCAATTGAATTACTTCGGTGCCGTTAGATTGGTCTTGAATTTGTTGCCACA<br>CATGATCAAGAGAAAGAACGGTCAAATTATCAACATCTCCTCCATTGGTGTTTTGGCTAATGCTACTAGATTCTCTGCTTACGTTGCTTCTAAAGCTGCTTTGG<br>ATGCTTTCTCTAGATGTTTGTCTGCCGAAGTTTTGAAGCACAAGATTTCTATTACCTCCATCTACATGCCATTGGTTAGAACTCCAATGATTGCTCCAACTAAG<br>ATCTACAAGTACGTTCCAACTTTGTCTCCAGAAGAAGCTGCTGATTTGATCGTTTACGCCATTGTCAAAAGACCAACTAGAATTGCTACCCATTTGGGTAGATT<br>GGCTTCTATTACTTACGCTATTGCCCCAGATATCAACAACATTTTGATGTCCATCGGTTTCAACTTGTTCCCATCTTCTACTGCTGCTTTGGGTGAACAAGAAA<br>AGTTGAACTTGTTGCAAAGAGCCTACGCTAGATTATTTCCAGGTGAACATTGGTGA

AsFACR

ATGAATGCCAAGTTGAAGAAGTTGTTCCAACAAAAGGTTGACGGTAAGACCATTATAGTTACTGGTGCTTCTTCCGGTATTGGTTTGACTGTTTCTAAGTATTT
GGCTCAAGCTGGTGCTCATGTTTTGTTGTTGGCTAGAACAAAAGAAAAGTTGGATGAAGTCAAGGCCGAAATTGAAGCTGAAGGTGGTAAAGCTACTGTTTTTC
CATGCGATTTGAACGATATGGAATCCATTGATGCCGTTTCCAAAGAAATTTTGGCTGCCGTTGATCACATCGATATCTTGGTTAACAACGCTGGTAGATCCATT
AGAAGAGCTGTTCATGAATCCGTTGATAGATTCCACGATTTCGAAAGAACCATGCAATTGAATTACTTCGGTGCCGTTAGATTGGTTTTGAATGTTTTGCCACA
CATGATGCAAAGAAAGGATGGTCAAATTATCAACATCTCCTCCATTGGTGTTTTGGCTAATGCTACTAGATTCTCTGCTTACGTTGCTTCTAAAGCTGCTTTGG
ATGCTTTCTCTAGATGTTTGTCTGCTGAAGTTCATTCCCATAAGATTGCTATTACCTCCATCTACATGCCATTGGTTAGAACTCCAATGATTGCTCCAACTAAG
ATCTACAAGTACGTTCCAACTTTGTCTCCAGAAGAAGCTGCTGATTTGATTGCTTACGCCATTGTCAAAAGACCAAAGAAGATTGCTACCAACTTGGGTAGATT
GGCTTCTATTACTTACGCTATTGCCCCAGATATCAACAACATTTTGATGTCCATCGGTTTCAACTTGTTCCCATCTTCTACTGCTTCAGTTGGTGAACAAGAAA
AGTTGAACTTGATCCAAAGAGCCTACGCTAGATTATTTCCAGGTGAACATTGGTGA

SeFAAR

ATGTTTGGTTTGATCGGTCATTTGACATCCTTGGAACAAGCTAGAGATGTCTCTAGAAGAATGGGTTATGATGAATACGCCGATCAAGGTTTGGAATTTTGGTC
ATCTGCTCCACCACAAATCGTTGACGAAATTACTGTTACTTCTGCTACCGGTAAGGTTATCCATGGTAGATATATTGAATCCTGCTTCTTGCCAGAAATGTTGG
CTGCTAGAAGATTCAAAACTGCTACCAGAAAGGTTTTGAACGCTATGTCTCATGCCCAAAAACATGGTATTGATATTTCTGCTTTGGGTGGTTTCACCTCCATT
ATTTTCGAAAACTTCGATTTGGCCTCCTTGAGACAAGTTAGAGATACCACTTTGGAATTCGAAAGATTCACTACTGGTAACACTCATACCGCTTACGTTATCTG
TAGACAAGTTGAAGCTGCTGCTAAGACTTTGGGTATTGATATCACTCAAGCTACCGTTGCTGTTGTTGGTGCTACTGGTGATATTGGTTCTGCTGTATGTAGAT
GGTTGGATTTGAAATTGGGTGTTGGTGATTTGATATTGACCGCCAGAAATCAAGAAAGATTGGATAACTTGCAAGCCGAATTGGGTAGAGGTAAAATTTTGCCA
TTGGAAGCTGCTTTGCCAGAAGCTGATTTTATCGTTTGGGTTGCTTCTATGCCACAAGGTGTTGTTATTGATCCAGCTACTTTGAAGCAACCATGCGTTTTGAT
TGATGGTGGTTACCCAAAGAACTTGGGTTCTAAAGTTCAAGGTGAAGGTATCTACGTTTTGAATGGTGGTGTTGTTGAACACTGCTTCGATATTGATTGGCAAA
TTATGTCCGCTGCTGAAATGGCTAGACCAGAAAGACAAATGTTTGCTTGTTTCGCTGAAGCCATGTTGTTGGAATTTGAAGGTTGGCATACCAATTTCTCTTGG
GGTAGAAATCAAATCACCATCGAAAAGATGGAAGCTATTGGTGAAGCCTCTGTTAGACATGGTTTTCAACCATTGGCTTTGGCCATTTGA

AvFAAR

ATGTTTGGACTAATTGGTCATCTAACTTCATTGGAACATGCCCAGGCTGTTGCTCAGGAACTAGGTTACCCAGAATACGCAGATCAGGGTCTTGACTTTTGGTG
TTCTGCTCCACCGCAAATAGTCGACCACATCAAGGTCACTTCTATTACCGGTGAAATAATTGAAGGTCGTTACGTCGAGAGTTGTTTTTTACCAGAGATGTTAG
CCAGCAGACGTATTAAAGCTGCTACTAGAAAAGTATTGAACGCCATGGCGCACGCACAAAAGCATGGTATTGATATTACCGCACTGGGGGGCTTTTCTTCTATA
ATCTTTGAGAATTTTAAATTGGAACAATTCAGTCAAGTCCGTAATGTTACCCTAGAGTTTGAAAGATTTACTACTGGGAATACACACACTGCCTACATAATATG
CAGGCAAGTTGAACAGGCTAGTCAGCAACTGGGCATTGAATTATCCCAAGCAACAGTTGCAATCTGTGGGGCTACAGGAGATATTGGTAGCGCAGTTACAAGAT
GGTTGGACGCTAAGACTGACGTTAAAGAACTACTGTTGATAGCCAGGAATCAAGAAAGACTTCAAGAGTTACAAAGTGAATTGGGAAGAGGTAAAATTATGTCC
CTGGACGAGGCCCTGCCACAGGCAGATATAGTCGTTTGGGTGGCATCAATGCCTAAAGGTGTCGAAATAAATCCTCAAGTGCTAAAGCAACCATGTCTATTGAT
AGATGGGGGCTATCCCAAGAATTTAGGAACTAAAGTGCAGTATCCCGGGGTTTATGTCTTGAATGGGGGTATAGTGGAACACAGCCTAGATATTGATTGGAAGA
TCATGAAAATTGTTAACATGGATGTGCCTGCTAGACAGTTATTTGCTTGCTTTGCAGAATCCATGTTATTAGAGTTTGAAAAATTATATACCAACTTTAGCTGG
GGTAGAAATCAAATTACCGTCGATAAAATGGAACAAATCGGCCAAGCCAGTGTTAAACATGGTTTCAGGCCGTTATTAGTATGA

ApFAAR

ATGTTTGGTTTAATAGGTCATTTGACTTCCTTAGAACACGCACAAGTAGTAGCCAGAGATTTGGGTTACGCAGAATACGCAGATCAAGGTTTGGATTTTTGGTG
TTCTGCTCCACCTGTAATTGTTGAAGACTTAAAGGTTACATCAATCACCGGTCAAGTCATTGAAGGTAGATATGTAGAATCCTGCTTCTTGCCAGAAATGTTAG
CTACTAATAGAATGAAGGCTGCAACAAGAAAGATTATAAACGCAATGGCCCATGCTCAAAAGAATGGTATTAACATCACTGCATTGGGTGGTTTTTCTTCAATC
ATTTTAGAAAGATTCAATTTGGATCAATTGGGTAGAATCAGAAACATCAAGTTGGAATTCGAAAGATTCACTACAGGTAATACCCACACTGCTTACATAATCTG
TAGACAAGTTGAACAAGCCGCTCCTAAATTGGGTATTGATTTGTCAAAGGCAACCGTCGCCGTATGTGGTGCAACTGGTGACATTGGTTCCGCCGTATGCAGAT
GGTTGAATGGTAGATTGGATGTTGCCGAAATCTTGTTGATCGCTAGAGACAGACAAAGATTGCAAAACTTACAAGCCGAATTGGGTAGAGGTAAAATCATGGCC
TTGGATGAAGCATTGCCAAAGGCAGACATTGTTGTCTGGGTCGCATCTATGCCTCAAGGTGTTGAAATAGATCCAGAAGTCTTGAAGAAACCTTGTTTGTTAAT
TGACGGTGGTTACCCTAAAAATATGGCTACAAAGTTCCAATCCCCTGGTGTTCATGTCTTGAGTGGTGGTATAGTTGAACACGCATTGGATATCGACTGGAAGA
TCATGAAGATCGTAAACATGAACGTTCCAGGTAGACAATTGTTTGCTTGCTTCGCAGAAAGTATGTTGTTAGAATTCGAAGCTATCTATACAAACTTCTCTTGG
GGTAGAAACCAAATAACCTTAGATAAGATGGATATGATTGGTAGAATGAGTATTAAGCACGGTTTCAAGCCTTTGATGTTGTGA

Table S7. ALK production by BY4741/pTT-ALK

ALK	Titer ($\mu g\ L^{-1}$)
Tridecane (C13:0)	2.8 ± 1.4
Pentadecane (C15:0)	101.5 ± 6.4
7-Pentadecene (C15:1)	9.8 ± 0.5
Heptadecane (C17:0)	111.7 ± 0.5
8-Heptadecene (C17:1)	16.2 ± 0.5

Table S8. Aldehyde production by BYΔ6H/pmaFACR$_{SYK}$

**Aldehyde**	**Titer (µg L^{-1})**
Hexadecenal (C16:0)	19.1 ± 1.8
9-Hexadecenal (C16:1)	ND
Octadecanal (C18:0)	13.9 ± 4.9
9-Octadecenal (C18:1)	ND

ND – below detection limit

4 References

Dicarlo, J.E., Norville, J.E., Mali, P., Rios, X., Aach, J., and Church, G.M. (2013). Genome engineering in Saccharomyces cerevisiae using CRISPR-Cas systems. *Nucleic acids research* 41**,** 4336-4343. doi: 10.1093/nar/gkt135.

Engler, C., Gruetzner, R., Kandzia, R., and Marillonnet, S. (2009). Golden gate shuffling: a one-pot DNA shuffling method based on type IIs restriction enzymes. *PloS one* 4**,** e5553. doi: 10.1371/journal.pone.0005553.

Foo, J.L., Susanto, A.V., Keasling, J.D., Leong, S.S., and Chang, M.W. (2017). Whole-cell biocatalytic and de novo production of alkanes from free fatty acids in Saccharomyces cerevisiae. *Biotechnol. Bioeng.* 114**,** 232-237. doi: 10.1002/bit.25920.

Heigwer, F., Kerr, G., and Boutros, M. (2014). E-CRISP: fast CRISPR target site identification. *Nat. Methods* 11**,** 122-123. doi: 10.1038/nmeth.2812.

Jakociunas, T., Bonde, I., Herrgard, M., Harrison, S.J., Kristensen, M., Pedersen, L.E., Jensen, M.K., and Keasling, J.D. (2015). Multiplex metabolic pathway engineering using CRISPR/Cas9 in Saccharomyces cerevisiae. *Metabolic engineering* 28**,** 213-222. doi: 10.1016/j.ymben.2015.01.008.
